# Pain Assessment for Individuals with Advanced Dementia in Care Homes: A Systematic Review

**DOI:** 10.3390/geriatrics6040101

**Published:** 2021-10-19

**Authors:** Nansi Felton, Jennifer S. Lewis, Sarah-Jane Cockburn, Margot Hodgson, Shoba Dawson

**Affiliations:** 1 Avon and Wiltshire Mental Health Partnership NHS Trust, Bath NHS House, Newbridge Hill, Bath BA1 3QE, UK; S-J.Cockburn@soton.ac.uk (S.-J.C.); margot.h.hodgson@gmail.com (M.H.); 2 School for Health and Social Wellbeing, University of the West of England, Bristol BS16 1DD, UK; Jenny4.Lewis@uwe.ac.uk; 3 National Complex Regional Pain Syndrome Service, Pain Specialty, Royal United Hospitals NHS Trust, Bath BA1 3NG, UK; 4 Doctoral College, Department of Psychology, University of Southampton, Building 44, Highfield Campus, Southampton SO17 1BJ, UK; 5 Bristol Medical School, University of Bristol, Bristol BS8 2PS, UK; shoba.dawson@bristol.ac.uk

**Keywords:** pain assessment, advanced dementia, care homes, systematic review

## Abstract

Pain is prevalent in older people, especially in those with advanced dementia who have communication impairments. Although pain is recognised to be present in this population, it is often under-assessed and ineffectively managed. The assessment of pain in advanced dementia is extremely challenging and complex, particularly in institutional settings such as care homes. This study systematically reviews the literature to examine and characterise the evidence for the use of pain assessment tools in care homes with individuals living with advanced dementia. Relevant publications were sourced from electronic bibliometric medical databases including AMED, CINAHL Plus, Medline, PsycINFO, EMBASE, TRIP Pro, Google Scholar, and HINARI. The database search was supplemented by screening citations and reference lists, in addition to a grey literature searches. The search identified 2221 studies, among which 26 were included in the review. The majority of the studies were observational, which created a rich source of data to create four major themes. The findings were informed and shaped by working with key stakeholders to develop a conceptual model that can contribute to developing evidence-based practice. This highlights the importance of a comprehensive, multi-disciplinary approach to pain assessment in this population, which is beyond the use of tools.

## 1. Introduction

Pain in older adults is prevalent and often underestimated and under-treated, especially in institutional settings such as care homes. Individuals living with dementia are more at risk of pain being undetected, particularly as the condition progresses into the later stages. Individuals with advanced dementia who have limited ability or reliability to self-report pain makes the assessment even more challenging and complex [1,2,3,4,5]. A large number of observational pain tools have been developed based on the behavioural cues to identify potential pain. There has been a relatively large number of evaluations and reviews of such pain assessment tools, although there is no one tool that is recommended for use in this population [6,7]. In addition, the care home setting has unique challenges and difficulties in how to integrate pain assessment tools into practice due to the complex care needs of the residents, the high staffing turnover, and the budget considerations [8,9,10].

In an ever-increasing older population, with a predicted increase in 8.2 million people over 65 living in the UK in 50 years’ time [11], the number of individuals living with dementia is set to rise and dementia has been recognised as a public health priority. Furthermore, about 70% of people in care homes have some type of dementia and about 80% of those are living with chronic pain [4]. This is therefore a highly relevant and important area of interest, particularly in relation to assessing pain in the later stages of dementia in a care home setting, which is the focus of this review.

### Aims of the Review

- To identify what pain assessment tools have been used to assess pain in advanced dementia care in care home settings globally including their psychometric properties; and
- To explore the implications of using pain assessment tools in practice through the medium of a narrative synthesis.

## 2. Materials and Methods

A systematic review was conducted and reported in accordance with the Preferred Reporting Items for Systematic Reviews and Meta-Analyses (PRISMA) guidelines [12,13]. A protocol was registered in PROSPERO CRD42019122239.

### 2.1. Search Strategy

A comprehensive search strategy was used including combinations of three main blocks of terms including and relating to ‘Dementia’ and ‘Pain Assessment’ and ‘Care Homes’ using a combination of Medical Subject Headings (MeSH) and free-text terms wherever relevant and possible (Table S1: Search Strategy). Electronic bibliometric medical databases including Medline, EMBASE, CINAHL, PsycINFO, and Cochrane library were searched for potential studies from inception to January 2019 (and then updated in June 2020 to include any recent and relevant studies as the initial searches were more than a year old). Additionally, citation searches and reference lists of included studies and systematic reviews, grey literature searches (TRIP Pro, Google Scholar, HINARI, and PROSPERO) supplemented the database searches. Search strategies were developed with support from a specialist librarian and several scoping exercises were run in different electronic databases to maximise the sensitivity and specificity of the developed search strategy.

### 2.2. Eligibility Criteria

Studies were included in the review if they met the following criteria:

Population: Adults aged 65 or over, diagnosed with dementia including the primary subtypes of dementias (such as Alzheimer’s disease, vascular, Lewy body, fronto-temporal) as diagnosed using any recognised criteria. If studies included a mixed population (early and later stages of dementia), studies were only included if at least 50% of the population included in the study had advanced dementia and in cases where this was unclear, it was excluded.

Intervention: Studies that described a pain assessment tool used for assessing pain in individuals with advanced dementia including sub-groups of dementia in older adults residing in care home settings were considered. Studies that did not include at least one recognized pain assessment tool were excluded.

Types of studies: All study designs were eligible for inclusion. 

Setting: Studies in care home settings including residential and nursing homes. Studies were included irrespective of country and language. Studies in secondary or tertiary care hospital setting were excluded.

Only studies published in the English-language were included in this review.

### 2.3. Study Selection

An Endnote library was used to combine and export the results of the searches from the different databases. Study selection was completed in two stages. First, NF independently screened titles and abstracts in order to identify eligible and relevant studies. A random sub-sample of 50% was screened by a second reviewer (SC/MH/SD) independently. As the rate of agreement was high, screening of the remaining titles and abstracts was continued by the primary researcher independently. Subsequently, full texts of the relevant studies were screened and reviewed in full by NF for eligibility and a random subset sample of 30% were screened independently by a second reviewer (SC/MH/SD). At both stages, any disagreements were resolved through discussions.

### 2.4. Data Extraction and Critical Appraisal

A data extraction form was devised in Microsoft Word and piloted on 20% of randomly selected studies. It was then checked by a second reviewer (MH) and a third reviewer (SD). Data for the remaining studies were extracted by one reviewer (NF) and double checked by JL and SD. The following descriptive data for the included studies were extracted:

- Study characteristics: author, date and country;
- Participant characteristics: diagnosed with a dementia, living in a care home setting;
- Aims of the study;
- Sample size, age range;
- Methodology: study collection, data collection process, synthesis of results, risks of bias;
- Pain assessment tool/s used;
- Main findings; and
- Recommendations made.

### 2.5. Quality Assessment

Assessment was carried out in the study selection to assess the strengths and weaknesses of each study. Due to the heterogeneous nature of the included studies, a single quality assessment tool was used—the Mixed Methods Appraisal Tool (MMAT)—to appraise quantitative, qualitative, and mixed method studies [14]. Quality appraisal was completed by NF and checked by JL/SD. Any discrepancies were resolved through discussions. Quality in terms of the reliability and validity of the pain assessment tools were evaluated using a tool developed by Zwakhalen et al. [15] to evaluate (1) the origin of the items; (2) number of participants; (3) content validity (the items cover all pain dimensions); (4) validity of criteria; (5) validity of construct I (in relation to another pain scale); (6) validity of construct II (difference between pain/no pain); (7) homogeneity (Cronbach’s alpha); (8) reliability between observers; (9) test–retest reliability; and (10) applicability. The total score can range from 0 to 20 for each instrument. This was used in a recent study to explore pain assessment in this population in Latin America [16]. 

## 3. Results

### 3.1. Search Results

**Figure 1:**
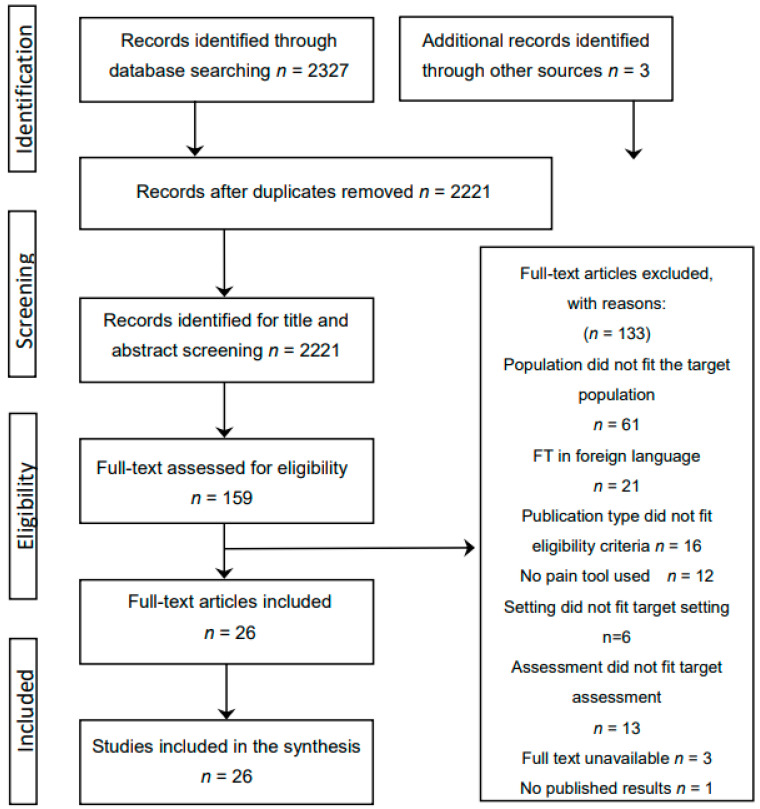
Search strategy using the PRISMA flowchart.

In total, 2327 titles and abstracts were identified from searching the electronic databases and screened for eligibility. This created a source of 2221 studies after duplicates were removed (*n* = 106), for title/abstract screening. After reviewing titles and abstracts, this was reduced to 159 for full text screening, after which a total of *n* = 23 individual studies were included. Three additional studies were identified through citation searches, searching the reference lists, and grey literature, with a total of *n* = 26 studies included in the narrative synthesis (Figure 1).

### 3.2. Summary of the Studies

The majority of the studies were observational (15 out of the 26), which was expected as the review question explores observational scales used to assess pain in a population who cannot reliably verbalise their pain. In addition, there were two cross-sectional studies, one longitudinal study, five evaluation studies, and three cluster RCTs. 

Studies were conducted in the Netherlands (*n* = 5), the United States of America (*n* = 4), Australia (*n* = 4), Norway (*n* = 4), Canada (*n* = 3), the UK (*n* = 2), Italy (*n* = 2), Poland (*n* = 1), and New Zealand (*n* = 1). The sample sizes also varied, ranging from 10 to 352, with a mean size of 117. An additional strength was that most of the studies used clinical staff as pain assessors who were familiar with the participants, which is fundamental to person-centred care and therefore a much more accurate assessment (Table 1).

The literature shows that a large number of observational pain assessment tools have been developed; this review identified 17 pain assessment scales (including different versions of scales) used worldwide in care home settings for those with advanced dementia (Table 2).

The most frequently used tools are PACSLAC (including the three variations) and PAINAD, each used in the seven included studies. The choice of these tools recognises them as international scales with good psychometric qualities and clinical utility, developed by experienced dementia care clinicians for this population [29,39,41,42]. MOBID and MOBID2 were used in five of the selected studies; scales that were also developed for this population with some established validity [21,23]. The Abbey Pain Scale was only used in three studies, which may reflect its lack of validity and internal reliability [4,35]. It is, however, used widely in the UK and Australia as a useful, easy to use clinical device and is recommended by the Australian Pain Society [4,34]. Two of the three studies that selected APS were Australian and so suggests an element of selective bias based on location. 

### 3.3. Quality Appraisal

A summary of the quality appraised studies included is shown in a data extraction form (Table S2: Data Extraction Form). Using the MMAT tool, each study was allocated an appropriate assessment using the five criteria with a ‘yes’, ‘no’, or ‘unsure’ scoring system. Findings were reported in a detailed presentation in relation to the five criteria, providing a quality of information, as advised by the tool creators [14]. Applying a scoring system is acknowledged as an informative contribution to the appraisal process (Table 3).

The studies were generally of good quality, with the exception of two studies that were of low quality (Table 3), using appropriate research design and rigorous data analysis. A majority of the studies selected were observational, which are usually more susceptible to bias. However, appropriate methods and outcome measures were used in the studies to help reduce the risk of bias and account for various confounders. Aspects of quality that were lacking included the use of convenience sampling. This could result in selection bias or limit the generalisation of results. However, use of this sampling method could be justified to enable targeting of a specific population to suit the primary aim of the research. 

In the 26 studies, there were 17 pain assessment tools (including variations) identified as suitable for psychometric evaluation and were analysed for their reliability and validity (Table S3). 

Not all the studies included psychometric testing of the tools and so could not contribute to this data. Additionally, there was considerable gaps in the data, with further validity and reliability testing required. The tools that scored the highest were the PACSLAC, the Abbey Pain Scale, and the Mahoney Pain Scale each scoring 15/20. However, the score implies that the overall scores were moderate and could be improved. All scales require further testing to improve and refine them including complete validation and reliability studies and larger samples. Additionally, the authors leading the evaluation of the Mahoney Pain Scale had a personal interest in developing the scale. 

### 3.4. Data Synthesis

Due to the heterogeneous nature of the included studies, a narrative synthesis approach is appropriate to summarise the current state of knowledge in relation to the review question [43]. This approach brings together the findings from the included studies in order to draw conclusions about the available evidence. This further contributes to the findings of this systematic review, highlighting important characteristics of the pain assessment tools, their clinical use in this context, and the relevant impact and implications that could not be reported statistically. 

A narrative synthesis of the literature included taking an iterative approach to develop themes. This involved a preliminary synthesis, followed by identifying primary themes exploring relationships, similarities, and differences within and between studies [43].

#### 3.4.1. Preliminary Synthesis

A preliminary synthesis was developed using the tabulation of pain assessment tools, grouping studies, and looking for patterns in relation to the review question, and thematic-analysis of good quality primary data. This enabled identification of recurrent and key themes and concepts across studies.

**Figure 2:**
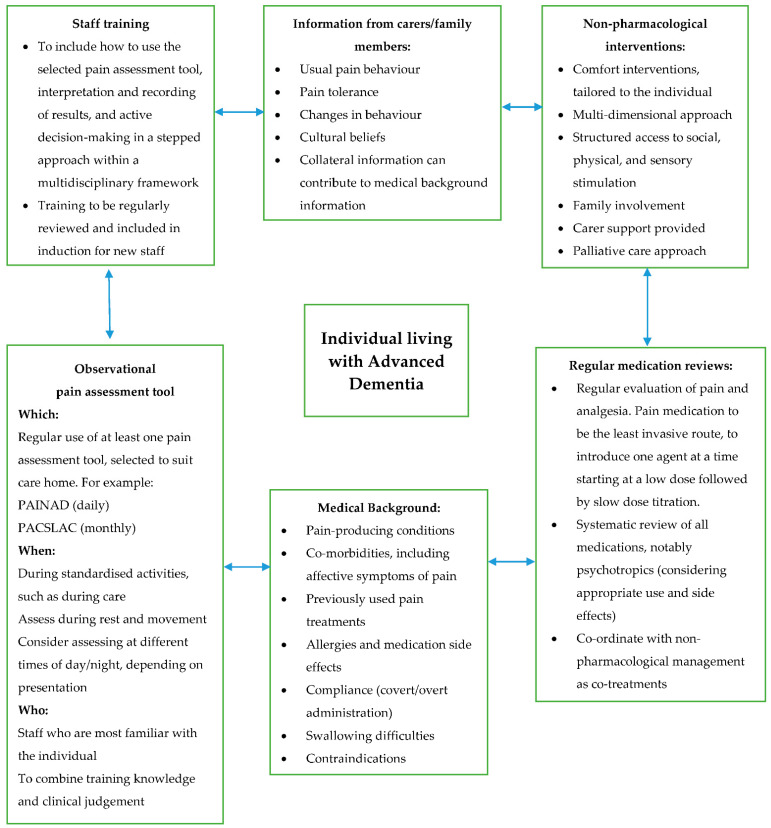
Conceptual Model. Integration of standardised and person-centred models of pain management for individuals living with advanced dementia in care homes.

However, no one tool is recommended, which contributes to the lack of consistent use of such tools in practice. A common reason is that there is no ‘gold standard’ pain assessment tool for this population [17]. This is in contrast to the advocated gold standard of self-report in pain assessment, which can be challenging or unreliable when assessing pain in the later stages of dementia. This is due to difficulties such as individuals often presenting with speech, expression, and interpretation of pain [44]. A number of key patterns have emerged from the literature, which are then represented in a conceptual model (Figure 2) of integrating standardised and person-centred models of pain management for this population developed in consultation with a variety of stakeholders. The characterisation of pain assessment tools and approaches used in this setting with this population also contributes to the examination of barriers, together with opportunities to improve clinical practice. 

**Figure 3:**
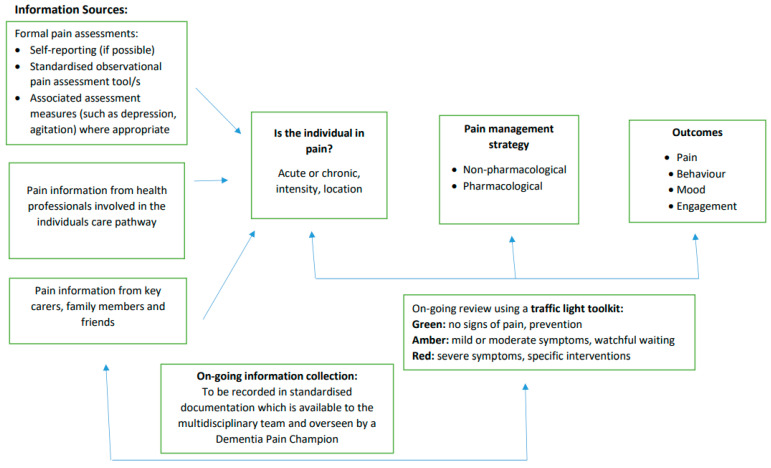
Pain Decision-making Model. Advanced dementia in the care home setting.

The review findings were informed and shaped by involving diverse stakeholders at the stage of synthesis and conceptual development. Stakeholder contributions included a Care Providers Forum, composed of commissioners and care home providers. Experts in the field were consulted for feedback on the Conceptual Model (Figure 2) and the Pain Decision-Making Model (Figure 3), which assisted in shaping them to their current form. Experts included members of the Dementia Health Integration Team (HIT), composed of academic and clinical specialists, together with expert patients and carers. Clinical specialists were also consulted in the form of Later Life Care Home Liaison Teams, specialised in dementia care. Stakeholders will be central to on-going dissemination of findings and trialling the model in practice.

The evidence-based Conceptual Model and Pain Decision-Making Model were therefore informed by the literature included in this review and shaped by key stakeholders from various academic, clinical, and public domains.

#### 3.4.2. Primary Themes

Four primary patterns, or themes, emerged from the literature: behavioural indicators of pain, staff training and education, affective symptoms of pain, and the multi-dimensional aspects of pain. These are summarised below in relation to the literature findings.

#### Behavioural Indicators

The detection of pain in individuals with advanced dementia presents unique challenges associated with the reduced ability to comprehend information and reliably communicate pain. In this group, behavioural indicators provide more reliable signs of pain than using the ‘gold standard’ of pain assessment, which is self-reporting. A number of pain assessment tools have been developed that include behavioural indicators of pain, which apply to people with dementia. These pain tools are predominantly based on guidance published by on the American Geriatrics Society categorisation of six domains for pain assessment, as listed below:

The six behavioural domains of pain (American Geriatrics Society Guidelines 1998, updated in 2002 and 2009 [45]):

- Facial expressions such as grimacing;
- Verbalisations and vocalisations such as groaning;
- Body movements, such as rocking;
- Changes in interpersonal interactions such as aggression;
- Changes in activity patterns and routines such as sleep; and
- Changes in mental state such as confusion.

Despite the most recent update of these guidelines being in 2009, they remain relevant to current approaches to assessing pain in this population. They are recognised as the basis to observational pain assessment tools, as reflected in more recent studies and guidelines [1,6,46]. However, it is also recognised that the interpretation of these behaviours is complex when applied to dementia. There may be overlap with common behavioural symptoms associated with dementia, and there may be some domains of pain more relevant than others to this group. These are explored as part of this review, particularly in relation to the importance of facial expressions. 

National guidelines on pain management in older people promote the use of standardised assessment tools using pain-related behaviours [4]. Procedures involved in caring for individuals in a care home are often standardised, and using such an approach to pain assessment can ensure it is not overlooked. However the research suggests that such guidelines and assessment tools are not being adhered to. Furthermore, the literature reflects the lack of clarity regarding the frequency of assessment, when to assess, and which scale to use; there is especially a paucity of clear clinical guidance on pain assessment for this population in the care home setting.

A study by Cohen-Mansfield (2008) showed that when pain is identified using observational assessment tools, it leads to an increase in the administration of analgesia, which results in a reduction in pain. This was a pioneering study in its focus on a population with advanced dementia in a care home setting. It had important clinical implications in demonstrating the clinical utility of observational tools in this population. It found an absence of correspondence between self-reporting (when applicable) and observational measures of pain, suggesting that self-reporting can be open to personal bias and environmental factors. Behavioural assessment of pain is therefore less susceptible to confounding variables. The findings are also reflected in a more recent study by Malara et al. (2016), which states that self-reporting alone is insufficient to assess pain in this population. Cohen-Manfield’s study was also weighted in favour of the PACSLAC tool, which is associated with the researchers and so may reveal a bias towards this tool. Additionally, the PACSLAC-D is supported in a RCT study by Pieper et al. (2018), finding the importance of behavioural identifiers for pain assessment in advanced dementia. 

Cohen-Manfield’s study also demonstrated that the identification of pain can impact positively on the social engagement of individuals with their environments and therefore contribute to their quality of life. Such factors would need to be further explored within a qualitative study approach. The results of this study present a standardised methodology that caregivers could utilise to detect pain and enable more effective treatment. However, a limitation of such tools is that localisation or type of pain cannot be determined and this was suggested as an area for future research. 

Behaviour indicators are explored further by Husebo (2009), suggesting that pain behaviours can help with the assessment of pain intensity, but assessment can be impacted by variables such as interpretation of the behaviours including who is doing the assessment. Pain assessment tools and guidelines follow general principles and need to be used in conjunction with critical thinking, clinical judgement, and experience [4]. The study was conducted using video uptake, which questions the potential to overestimate pain observations rather than using ‘real life’ situations. Additionally, the study does not explore the relationship between the caregivers and the participants including how they were selected, their knowledge of the individual, and how this may impact on the results. The intensity and nature of pain behaviours, rather than the number, could be considered important when entering the process of pain assessment and highlights the value of clinical judgement and knowledge of the individual. 

Such challenges of using pain behaviours as part of pain assessment in this population is explored in a European survey on the use of pain assessment tools, practices, guidelines, and policies [5]. Carers and health care professionals may not be confident about judging their observations or interpreting pain signals, which is, in essence, subjective in nature. Additionally, this can vary according to staff education, experience, and levels of expertise, which emerged as a theme in the literature.

##### Staff Training and Education

There appears to be discrepancies in the literature regarding whether assessing pain from behaviours is dependent on the level or standing of the assessor. 

A study by Nowak et al. (2018) in Poland considered the characteristics of pain and the variables that may impact on using the recommended observational pain tools. This includes the estimation of pain being dependent on the knowledge of caregivers. It compares a previous study that showed that less than 15% of individuals suffering with moderate to severe cognitive impairment were treated for pain, compared to the current study, which showed a higher percentage. This is suggested to correlate with enhanced efforts in Poland regarding the education of staff on pain diagnosis and treatment. However, the small sample size should be considered in relation to the generalisation of the findings. In addition, other variables and reasons for administration of analgesia need to be considered. 

The study contributes to the findings of this review that pain related behavioural disturbances can be misinterpretated as symptoms of dementia and the consequent inappropriate prescription of psychotrophic medication. Even when these individuals scored high on the Abbey Pain Scale, they were not receiving adequate analgesia. This also highlights a lack in staff training and awareness of the different indications and signs for pain in individuals who cannot verbalise or express their pain reliably. 

This is consistent with conclusions drawn in a feasibility study by Zwakhalen et al. (2012). Although there were high compliance rates in using the pain assessment observational scale, pain prevalence was 22%, which is low compared to other studies, and pain relieving interventions were not frequently applied. The identification of pain using an observation tool therefore did not necessarily result in an increase in interventions. 

This study used qualitative information drawn from staff questionnaires on using the scales. Staff identified difficulties in interpreting pain cues and believed that a score could be increased by factors such as distress or panic, although primary data from the interviews were limited, so reduces the credibility. The study was also limited by a small sample size (*n* = 22) and time span (six weeks) so restricts external validity and suggests that further research with an extended length and larger participant number is needed. However, this is acknowledged as a limitation and consequently presents this work as a preliminary study with emphasis on the testing feasibility and piloting of the procedure. The method includes the Medical Research Council framework for the evaluation of complex interventions, strengthening the multifactorial nature of such interventions. A pre-intervention interview was conducted to enable the gathering of key information such as pain policy and staff knowledge of the individual participants. Two further evaluation interviews were conducted to gather staff experiences on assessing pain; repeated measurements increase confidence in the findings and provide a stronger study design. The study contributes valuable findings to the argument that providing staff with pain assessment tools alone is not sufficient to change practice. The tools may not differentiate between pain and other constructs such as distress or panic, and they do not contribute to specific clinical decisions regarding how to reduce or treat pain. 

##### Affective Symptoms of Pain

This raises the question of if or how pain assessment tools can contribute to pain management decisions. Behavioural signs of pain can reflect other behaviours, which may be secondary to dementia. Dementia is frequently accompanied by behavioural and psychological symptoms of dementia (BPSD), and is shown to result in inappropriate prescription of psychotropic medication or inappropriate admissions to mental health inpatient units [44]. Nowak et al. (2018) assessed analgesic treatment in nursing homes to delineate the relationship between pain and behavioural and psychological symptoms of dementia. The study used observational tools to assess levels of pain and agitation. It found a relationship between pain and agitation, suggesting that pain can be an important underlying cause of behavioural disturbances in individuals with dementia. The study showed that individuals with major behavioural disturbances were prescribed more sedatives, and even though the Abbey Pain Scale scored high, this did not result in the administration of analgesia. Therefore, behaviours including agitation, aggression, anxiety, and depression, which may indicate that the individual is in pain, were not being considered during medication decisions. However, the limitations of this study compromise the findings: there was a heterogeneity of different analgesics, not necessarily for the treatment of pain, and choosing the Abbey Pain Scale lacks supporting evidence.

Villanueva et al. (2003) recognises the need to routinely assess pain in dementia as a clinical necessity. The approach is a person-centred, holistic one, which includes the consideration of pain behaviours as well as the impact pain has on the activities of daily living, social behaviour, and sleep patterns. The study goes on to explore the close association between pain and agitation, especially in the later stages of dementia and identified verbal agitation as the most consistently associated with pain. Pain is sometimes treated as agitation using antipsychotic medication. This study suggests that pain, agitation, and psychiatric symptoms are closely linked or simultaneously interpreted by care home staff, and that further research is required to better understand the relationship between these factors. The study has strong clinical applicability; identifying the value of care staff (rather than researchers) as assessors when using pain scales in the clinical setting, with good inter-rater reliability.

Van Kooten et al. (2017) studied pain and its pharmacological treatment in relation to dementia sub-type and severity. The study highlights the need to focus pain management on tailored approaches and regular adjustment to individual needs. It considers different types of pain in this population, as determined through findings of a physical examination and information from medical records. It presents greater external validity as it has a larger sample size (*n* = 199), although this is compromised by a low response rate. It also uses a combination of pain assessment tools, which can account for the complexities involved in pain assessment. 

The findings demonstrate that residents with more severe dementia experience pain more often and with greater intensities. However, it was found that residents still suffered pain even when on regularly scheduled analgesia, suggesting that this method does not necessarily reflect optimum pain treatment. Systematic medication reviews are needed for a more tailored treatment approach. This supports the findings of a study by Griffioen et al. (2017), which explored the use of opioids in care homes with the prevalence of pain in this population remaining high. Additionally, Van Kooten et al. (2017) highlight confounding variables that impact on pain assessment; pain complaints are often associated with comorbid depressive symptoms and attention should be paid to this coexistence of depressive symptoms when assessing and treating pain. This was the first study on pain type and its pharmacological treatment in relation to dementia severity. The findings build upon a previous study by Cohen-Mansfield (2008) that promoted a much wider use of analgesics in this population. Van Kooten et al. (2017) used MOBID-2 to reflect clinical practice, as this uses standardised movement during morning care and considers the use of a combination of pain measurements. However, this strength is compromised by the use of the researcher, rather than clinical staff, to judge the residents’ understanding of the pain scales and ability to communicate pain, which may introduce an element of bias. 

On the other hand, Jordan et al. (2011) explored the use of PAINAD and DisDAT for detecting pain in this population, with PAINAD not having been previously explored in a UK population with severe dementia. PAINAD has 92% sensitivity, suggesting that if a person with severe dementia is in pain, this tool is likely to detect it. However, the researcher also discusses the high levels of false positives (33%), so other potential causes of behavioural changes should be explored. This tool, and other similar observational tools, should therefore not be regarded as definitive in the detection of pain in this population. Psycho-social distress can be difficult to distinguish from pain, and this further highlights the complicated nature of pain assessment and how there are several variables and influences that need to be considered.

Additionally, there is a lack of consensus in the literature about which behaviour signs are valid indicators of pain and differentiating this with psychological symptoms such as fear or anxiety [44]. This has been explored in relation to a novel pain assessment tool, the ePAT [17,25]. Hoti et al. (2018) found that facial descriptors are valid indicators of pain for nonverbal individuals living with advanced dementia. Additionally ePAT was found to have strong clinimetric properties. It is understood that in this population, facial expressions become an essential component of communicating the existence of pain and they are more facially expressive than healthy subjects in pain. The study was limited by a small sample size and to a specific setting so that the application to other settings may be limited. The findings were supported by Atee et al. (2018), although the authors declared being shareholders to the intervention. However, ePAT demonstrated good reliability properties as shown by a variety of robust measures, and combined facial expression with clinical behaviour indicators to identify pain. Facial expression was also found to be most frequently observed in relation to pain in previous investigations [26,40]. Additional research to further evaluate the six behavioural domains of pain [45] in relation to this specific population would contribute to more effective and relevant pain assessment and management. 

##### Multi-Dimensional Assessment

Pain assessment in advanced dementia is therefore a highly complex process involving a multitude of factors that can impact the findings. Pain, being a subjective experience, means that a person-centred approach to pain assessment is essential. In addition, person-centred approaches are important when working with individuals with dementia [47]. In the literature, there is little evidence that such an approach is being used in models of pain assessment in this population in care homes. It suggests the need for an algorithm, a clinically usable tool to guide clinical decision-making in pain assessment and management.

Malara et al. (2015) identified a close relationship between pain and behavioural and psychological symptoms in residents with dementia. Pain is often communicated in behaviours that can be challenging and complex. It found chronic pain to be almost double in residents with dementia, and lack of a well-defined pain assessment tool and documentation to be obstacles to successful pain management. The study recommends the use of observational pain assessment tools, in combination with self-reporting, in a multi-dimensional assessment of pain. The assessments carried out by carers is a strength of the study, although it does not fully explore the relationship of the carer to the individual or how other professionals could contribute valuable input to the assessment. 

This was further explored in a cluster RCT by Rostad et al. (2018). The study found no overall effect of regular pain assessment when using an observational pain assessment tool on the pain scores or use of analgesia for individuals with severe dementia. The study methodology had a number of strengths including the randomisation of clusters (a cluster being a care home) and the raters being blinded, thus reducing bias. There were 16 clusters across four nursing homes in four counties in Norway. In addition, it was carried out in real-life conditions, with data collected by carers or nurses who would be involved in nursing home practice and who would know the individuals. This led to a discussion around the value of input and information that can be provided by such key practitioners and carers in this setting, as discussed earlier in this review. Assessing pain in older people is very complex, with considerations of co-morbidities, polypharmacy, and complex causes of pain. Further complexities are considered when assessing pain in individuals with severe dementia, and the importance of knowing the individual can be a key factor in assisting with this process. A standardised pain assessment tool cannot directly lead to clinical decision-making around pain, rather, it can contribute to and complement clinical judgement and knowledge applied by the staff. The pre-requisite to person-centred care in dementia is knowing the person including familiarity with the person’s patterns of behaviour and pain tolerance, and so pain assessment needs to be multi-dimensional and multi-disciplinary. 

This could be extended to the consideration of demographic information of both the staff/raters and the participants. Rostad et al. (2018) considered this as a limitation; information such as cultural background, professional qualification and training as well as the experience of the staff who completed the assessments should be considered as having an impact on interpretations and subjective assessment when using the assessment tool. Additionally, it came through in the literature that most of the studies had limited or no minority representation or consideration of culture and ethnicity and how this interacts with pain expression and interpretation [17,40]. 

A recent study by Ersek et al. (2020) used a multi-dimensional approach to pain assessment to characterise pain experiences in this population. It identified an improvement in pain management in this group, with most residents having mild and intermittent pain and the majority receiving at least one pain therapy in the last week. The study used a rigorous, multidimensional evaluation, which contributes to the strength of its findings. However the sample was small, and the evaluations were carried out by clinicians who did not provide on-going care to the residents enrolled on the study. Additionally, the study found only 3% of the participants had documented non-drug pain therapy. The study identified factors that may affect this low rate including some nondrug therapies being inappropriate to this group (such as CBT or mindfulness), there may be lack of availability, or perceptions that such interventions are ineffective or time consuming in this group. Further research is needed to explore such barriers to contribute to reductions in medication use. Pieper et al. (2018) supports the use of a stepwise, multidisciplinary approach to reduce pain in this population. It has strong external validity, with a large sample size, and six month cluster RCT study, with low risk of bias. Intervention involved a tailored approach, focusing on physical and psychosocial unmet needs, although this placed increased time pressures and the training needs on staff.

## 4. Discussion

### 4.1. Summary of Findings

The assessment of pain in advanced dementia is extremely challenging and complex, often resulting in under-treatment and poor pain management within the care home setting. This review reflects the growing awareness of the importance of detecting pain in this population with close associations with quality of life [4,44]. The review findings reflect the aims to identify the observational pain tools used in this population, and to explore the implications of using pain assessment tools in practice. This exploration has been strengthened by key stakeholder involvement.

Pain perception and interpretation is highly subjective and can be influenced by a number of variables. This was explored by narrative synthesis, finding key challenges, and barriers, which can affect clinical decision-making around pain in this setting and how such barriers may be addressed. These include the role of the pain assessor, when and how pain assessments are conducted, the training provided to staff to use pain tools, and if/how clinical decisions are then informed around pain management.

First, there was variation across studies regarding the chosen primary pain assessor(s); differences between using a researcher or a key carer/nursing staff from the home, or a combination (Table 1). This reflects the importance of assessor familiarity with the individual resident, which relates to the level of person-centred care provided and in turn may impact on the outcome. A cluster RCT [37], for example, involved the primary nurse who had clinical knowledge as well as familiarity with the resident to complete pain assessments during their everyday practice. Providing staff with pain assessment tools alone is not sufficient to change practice or improve pain management decision-making. Staff training and education is important to enhance pain recognition and treatment, combined with clinical judgement and familiarity with the individual [35]. Incorporating regular all-staff training to inform decision-making in a stepped approach is crucial to managing pain in this setting and incorporating it into daily practice. 

Social heterogeneity was also evident in the literature. Across the studies, demographic detail provided was basic; frequently limited to age and gender [18,22,27] and two with references to the percentage of Caucasians in the sample [21,25]. Information on factors such as ethnicity, education levels, and socio-economic background of participants or assessors is absent or minimal, which may impact on the expression, interpretation, and assessments of pain [16,17,37,40]. There is scope for further research to explore this in greater depth. Additionally, studies tended to evaluate people with dementia as a homogenous group, without exploring the experience of pain assessment methods to the individual being assessed. This contradicts the advocation of each person’s dementia ‘journey’ being unique and the most appropriate and comfortable approaches or methods used as part of the person-centred approach [48]. This could further inform future areas of research.

A large number of observational pain assessment tools have been developed and evaluated. A systematic review of systematic reviews in 2014 explored pain assessment tools in adults with dementia [6] and found that no one tool can be recommended given the available evidence. Our review updates these findings, and contributes further by focusing explicitly on advanced dementia in the care home setting. Additionally, the findings of the current study reflect those of a similar study, which evaluated pain assessment tools in patients with advanced dementia at the Latin America level [16]. The current study adds further to the literature, being on a global scale. 

The current review identified 17 tools used in advanced dementia worldwide in this setting, which were suitable for psychometric evaluation (Table S3). The Assessment of Pain in Older People: UK National Guidelines 2018 asserts that an observation pain scale should be used, and recommends PAINAD and Doloplus-2 for individuals with advanced dementia in terms of reliability and validity [4]. This recommendation is also reflected in guidelines for older persons with dementia in care home facilities in Canada in addition to the Abbey Pain Scale [3], a scale that remains popular in the UK but lacks supporting evidence [4]. A recommendation by an expert group that focused on nursing homes recommended PACSLAC, PAINAD, and NOPAIN, but the first two were the most clinically relevant [8]. Use of these recommended pain scales is reflected in the findings of this review, with PACSLAC and PAINAD being the most widely used in studies for this specific population and setting (Table 2). PACSLAC demonstrated the stronger psychometric properties and clinical benefits (Table S3). PACSLAC has good content validity with an extensive item collection that specifically relates to characteristics of pain in individuals with advanced dementia, incorporating a multidimensional assessment of pain [15]. PACSLAC is therefore a useful tool in practice for this population, although consisting of a 60-item checklist, it is long and may not be clinically applicable as a daily assessment tool [4,18]. PACSLAC did not demonstrate adequate validity coefficients as the test–retest was missing in the data and sample numbers were small. Additionally, further evidence for the use of the shorter, more refined versions of PACSLAC II and PACSLAC-D in this population is required [4,30]. PAINAD, alternatively, consists of only five items and so is more user-friendly, although its brevity compromises the strength of the internal consistency [30]. 

The review findings suggest that the overall quality of all the scales could be improved through further testing and in larger samples. There remains no ‘gold’ standard for assessing pain in this population, in recognition of the fact that the use of self-report is not always being possible or reliable in the more advanced stages of dementia. The review findings conclude that a standardised, systematic approach to pain assessment may not be appropriate in advanced dementia care, but this should rather compliment the clinical judgement and experience of the staff including their knowledge of the individual. Such familiarity with the individual during pain assessment is crucial and has been termed as the ‘silver’ standard [6]. Making use of different perspectives in assessment methods, notably the disciplinary perspectives and, importantly in this context, the value of the input from those who were familiar with the resident is crucial [27,41]. 

The reviewed studies used different research methods and objectives, therefore making comparisons between studies difficult. However, this provided a rich diversity of information and offered a comprehensive summary of pain assessment and management for those with advanced dementia in a care home setting, as represented in the Conceptual Model (Figure 2). This highlights the importance of a comprehensive approach to pain assessment that is beyond the use of tools and within the context of a multidisciplinary framework.

Given that the studies were conducted in a variety of countries raises questions around cultural and organisational differences between care homes. This would need further exploration in future studies. Additionally, there is variation in situational characteristics across the studies, which may also affect generalisability. Moreover, pain assessment outcomes could potentially be affected by the time of day, the length of the assessment, the immediate environment, how the individual is positioned, or who is present. However, the impact of such factors is difficult to assess without appropriate levels of information provided by individual studies and many of these variables cannot be controlled. Due to the nature of the studies, the level of detail in terms of situational characteristics was limited to the pain assessments being conducted during standardized activities, primarily during care. The time of day may vary, depending on the presentation of the individual, which highlights the benefit of combining a standardized pain assessment with a person-centred approach. Consideration of the clinical application and relevance of the pain assessment as well as the situational characteristics of pain assessment should also be accounted for [30]. This also emphasises the need for a person-centred approach and the value of input from those who know the individual to interpret behaviours, which may include family members. It is, however, challenging to combine with standardised approaches, as recommended in algorithms of best practice and robust guidelines. The co-morbidity and combination of pain and impaired cognition is in itself very complex, and requires more specific guidance for assessing and managing pain in advanced dementia in care home settings. This is conceptualized in a theoretical framework to support pain assessment decision-making in this setting. This Pain Decision-Making Model (Figure 3) is to be launched as a pilot study in care homes in the South Gloucestershire locality, South West England, with the support of local Care Home Liaison Teams.

### 4.2. Methodological Critique

Rigorous search methods were employed in order to provide a comprehensive search of relevant studies to the review question. A Narrative Synthesis Framework (Figure S1) was applied for clarity and robustness, enabling a comprehensive narrative synthesis to summarise the current state of knowledge in relation to the review question. Additionally, the focus of the research question being pain assessment meant that selected studies included a recognised observational pain assessment tool. The review included an evaluation of the psychometric properties of the pain assessment tools identified in the literature, although this was limited by the studies that included the relevant data together with the limitations of the subjective nature of psychometric evaluation. 

Patterns emerging between studies were identified using tabulation and concept mapping as visual methods to construct themes, groupings, and relationships between the studies, which is a methodological strength of this review. A limitation is that there was heterogeneity, which made comparing some study findings difficult, although this is discussed in relation to the review question and does suggest further research is required to investigate by locality, social, and ethnic groups. Another strength of this review is that key researchers in the field were contacted to source unpublished literature, or studies that had not yet been published, although no further studies that met the inclusion criteria were identified. Additionally, a key methodology strength was involving the stakeholder in conceptual development, which meant that the model is relevant to the population in practice. 

The search was restricted to the English language, so primary papers in other languages would have been excluded, but with no geographical restriction. The search included papers from a wide variety of countries, but a lack of data from low-income countries was apparent. This may be due to the language restriction. 

### 4.3. Implications for Practice

The present review highlights a number of key points from a practice perspective, which resonate on a global scale. First, there is an extensive number of observational pain assessment scales available; without evidence to recommend one particular scale or ‘gold standard’ pain assessment, which would support organisational decision-making. Second, a multidisciplinary approach to pain assessment in this population is essential, which is both structured and person-centred. Third, assessing pain in advanced dementia is extremely complex and challenging. It requires more than an assessment tool; the value of professional judgement, training, and experience together with familiarity with the individual in pain is crucial in the pain management of this population.

There needs to be efficient and standardised methods of eliciting and centralising pain related information for this group in the care home setting. This would provide the basis for making decisions about pain management and treatment. It therefore requires a conceptual shift in care home practices, incorporating the benefits of both standardised and person-centred approaches. The process would also require regular training to staff regarding the use of protocols, guidance, and pain assessment tools, framed by a multi-disciplinary approach. This in turn could reduce the levels of work-related carer stress [22]. 

To be able to achieve this in the care home setting is a challenge due to the complexities of the care needs of this population, the high staff turnover as well as cost restrictions [10]. An evidence-based pain protocol needs to be developed and trialled in care homes to standardise pain management while maintaining a person-centred approach. The conceptual framework developed from this review could be the basis for such a protocol. The challenge is presenting the findings into an approach that can be used by care home staff in practice. The traffic light system has been incorporated into the Pain Decision-Making Model (Figure 3) for this purpose. It is based on a toolkit devised by the Alzheimer’s Society UK to provide evidence-based guidance for health and social care with professional caring for people with dementia [49]. The amber ‘watchful waiting’ is particularly relevant to the findings of this review, with a number studies promoting the importance of regular reviews, assessments, and the provision of non-pharmacological interventions to optimise the individual’s comfort and quality of life. This model needs future testing in practice to evaluate and develop it clinically.

There is also growing interest and evidential support for use of the ePAT [17,25]. Facial descriptors are used in many observational pain assessment tools, but this is the first tool to use automation. It has been found that individuals with dementia are more facially expressive, and the ePAT has strong clinometric properties. However, the studies selected in this review were limited to Australia with small sample sizes and so application to other locations may be restricted [17,25]. However, this is a promising area of research, reflected in current work on facial-recognition technology to identify pain in severe dementia for individuals in care homes [50]. Further research could potentially assist clinical practice in this particular area.

## 5. Conclusions

The assessment of pain in individuals with advanced dementia is complex and challenging for care home staff to manage. This review highlights the current state of the evidence base in this area including the gaps in knowledge and the implications for practice.

This review shows the importance of a comprehensive approach to pain assessment in care homes for this population, which is beyond the use of assessment tools. A holistic, multi-disciplinary approach is essential, which is both structured and person-centred. Pain tools complement clinical information and clinical judgement together with familiarity with the individual. The pain assessment process needs to be on-going and supported by staff training. There is a profound lack of specific guidelines to assist with decision-making in this setting to support this very vulnerable population. Therefore, there needs to be further research to consider the perspective of the individual, who is central to the process.

## Figures and Tables

**Table 1:** Summary of the studies.

Study ID	Location	Pain Assessment Tool/s Used	Type of Study	Pain Assessor	
				Clinical Staff	Researcher
Atee et al. 2018 [17]	Australia	Electronic Pain Assessment Tool [**ePAT**]	Observational	√	
Cheung et al. 2008 [18]	New Zealand	Pain Assessment Checklist for Seniors with Limited Ability to Communicate [**PACSLAC**]	Observational	√	√
Cohen-Mansfield et al. 2006 [19]	USA	Checklist of Non-verbal Pain Indicators Scale [**CNPI**] Non-communicative Patient’s Pain Assessment Instrument [**NOPAIN**] Pain Assessment for the Dementing Elderly [**PADE**] Pain Assessment in Advanced Dementia [**PAINAD**] Pain Assessment in Non-communicative Elderly [**PAINE**]	Evaluation	√	
Cohen-Mansfield et al. 2008 [20]	USA	**CNPI** **PADE** **PAINAD** **PAINE**	Evaluation		√
Ersek et al. 2020 [21]	USA	Mobilisation-Observation-Behavioural—Intensity-Dementia [**MOBID**] Pain Intensity Measure for Persons with Dementia [**PIMD**]	Evaluation	√	√
Fuchs-Lacelle et al. 2008 [22]	Canada	**PACSLAC**	Longitudinal study	√	
Griffioen et al. 2017 [23]	Norway	**MOBID-2**	Cross-sectional	Not stated	Not stated
Hadjistavropoulos et al. 2018 [24]	Canada	Facial Action Coding System [**FACS**] **PACSLAC-II**	Observational		√
Hoti et al. 2018 [25]	Australia	Abbey Pain Scale [**APS**] **ePAT**	Observational	√	√
Husebo et al. 2009 [26]	Norway	**MOBID**	Observational	√	
Husebo et al. 2014 [27]	Norway	**MOBID-2**	Cluster RCT	√	
Jordan et al. 2011 [28]	UK	**PAINAD**	Observational	√	√
Jordan et al. 2011 [29]	UK	Disability Distress Assessment Tool [**DisDAT**] **PAINAD**	Observational	√	√
Lints-Martindale et al. 2012 [30]	Canada	Assessment of Discomfort in Dementia [**ADD**] **CNPI** **PACSLAC PADE PAINAD** Non-communicative Patients’ Pain Assessment Instrument [**NOPAIN**]	Observational		√
Mahoney et al. 2008 [31]	Australia	Mahoney Pain Scale [**MPS**]	Evaluation	√	
Malara et al. 2016 [32]	Italy	**PAINAD**	Observational	√	
Monacelli et al. 2013 [33]	Italy	**DOLOPLUS-2**	Observational	√	
Neville et al. 2014 [34]	Australia	**APS** **CNPI** **DOLOPLUS-2**	Observational	√	
Nowak et al. 2018 [35]	Poland	**APS**	Observational	√	
Pieper et al. 2018 [36]	The Netherlands	Pain Assessment Checklist for Seniors with Limited Ability to Communicate-Dutch version [**PACSLAC-D**]	Cluster RCT	√	
Rostad et al. 2018 [37]	Norway	**DOLOPLUS-2**	Cluster RCT	√	
Van Dalen-Kok et al. 2019 [38]	The Netherlands	Pain Assessment in Impaired Cognition [**PAIC**]	Observational	√	
Van Kooten et al. 2017 [39]	The Netherlands	**PAINAD** **MOBID-2**	Cross-sectional	n/a	n/a
Villanueva et al. 2003 [40]	USA	**PADE**	Evaluation	√	
Zwakhalen et al. 2008 [41]	The Netherlands	**PACSLAC-D**	Observational	√	
Zwakhalen et al. 2012 [42]	The Netherlands	**PACSLAC-D**	Observational	√	

**Table 2:** Frequency of pain assessment tools.

Study ID	PACSLAC (II, D)	PAINAD	MOBID and MOBID2	CNPI	APS	DOLOPLUS-2	PADE	e-PAT	ADD	DisDAT	FACS	MPS	NOPAIN	OPBAI	PAIC	PAINE	PIMD
Atee et al. 2018 [17]								√									
Cheung et al. 2008 [18]	√																
Cohen-Mansfield et al. 2006 [19]		√		√													
Cohen-Mansfield et al. 2008 [20]		√		√			√							√		√	
Ersek et al. 2020 [21]			√														√
Fuchs-Lacelle et al. 2008 [22]	√																
Griffioen et al 2017 [23]			√														
Hadjistavropoulos et al. 2018 [24]	√										√						
Hoti et al. 2018 [25]					√			√									
Husebo et al. 2009 [26]			√														
Husebo et al. 2014 [27]			√														
Jordan et al. 2011 [28]		√															
Jordan et al. 2011 [29]		√								√							
Lints-Martindale et al. 2012 [30]	√	√		√			√		√				√				
Mahoney et al. 2008 [31]												√					
Malara et al. 2016 [32]		√															
Monacelli et al. 2013 [33]						√											
Neville et al. 2014 [34]				√	√	√											
Nowak et al. 2018 [35]					√												
Pieper et al. 2018 [36]	√																
Rostad et al. 2018 [37]						√											
Van Dalen-Kok et al. 2019 [38]															√		
Van Kooten et al. 2017 [39]		√	√														
Villanueva et al. 2003 [40]							√										
Zwakhalen et al. 2008 [41]	√																
Zwakhalen et al. 2012 [42]	√																
TOTAL	7	7	5	4	3	3	3	2	1	1	1	1	1	1	1	1	1

**Table 3:** Quality of studies analysis using the Mixed Method Appraisal Tool (MMAT) score.

**Study ID**	**Screen**		**Quantitative Randomised Controlled Trials**					**Quantitative Non-Randomised Studies**					**Total**
	S1	S2	2.1	2.2	2.3	2.4	2.5	3.1	3.2	3.3	3.4	3.5	Y %
Atee et al. 2018 [17]	Y	Y						Y	Y	N	Y	Y	4 high
Cheung et al. 2008 [18]	Y	Y						Y	Y	Y	Y	U	4 high
Cohen-Mansfield et al. 2006 [19]	Y	Y						Y	Y	Y	Y	U	4 high
Cohen-Mansfield et al. 2008 [20]	Y	Y						Y	Y	Y	Y	Y	5 high
Ersek et al. 2020 [21]	Y	Y						Y	Y	Y	Y	U	4 high
Fuchs-Lacelle et al 2008 [22]	Y	Y						Y	Y	Y	Y	Y	5 high
Griffioen et al. 2017 [23]	Y	Y						Y	Y	Y	Y	U	4 high
Hadjistavropoulos, et al. 2018 [24]	Y	Y						Y	Y	U	Y	Y	4 high
Hoti et al. 2018 [25]	Y	Y						Y	Y	Y	Y	Y	5 high
Husebo et al. 2009 [26]	Y	Y						Y	Y	N	Y	Y	4 high
Jordan et al. 2011 [28]	Y	Y						Y	Y	Y	N	Y	4 high
Jordan et al. 2011 [29]	Y	Y						Y	Y	Y	N	Y	4 high
Lints-Martindale et al. 2012 [30]	Y	Y						Y	Y	N	Y	Y	4 high
Mahoney et al. 2008 [31]	Y	Y						Y	Y	N	N	U	2 low
Malara et al. 2016 [32]	Y	Y						Y	Y	Y	Y	U	4 high
Monacelli et al. 2013 [33]	Y	Y						Y	Y	U	N	U	2 low
Neville et al. 2014 [34]	Y	Y						Y	Y	Y	Y	U	4 high
Nowak et al. 2018 [35]	Y	Y						Y	Y	Y	Y	Y	5 high
Van Dalen-Kok et al. 2019 [38]	Y	Y						Y	Y	N	Y	Y	4 high
Van Kooten et al. 2017 [39]	Y	Y						Y	Y	Y	Y	Y	5 high
Villanueva et al. 2003 [40]	Y	Y						Y	Y	Y	N	Y	4 high
Zwakhalen et al. 2008 [41]	Y	Y						Y	Y	Y	Y	N	4 high
Zwakhalen et al. 2012 [42]	Y	Y						Y	Y	N	Y	Y	4 high
Husebo et al. 2014 [27]	Y	Y	Y	Y	Y	Y	U						4 high
Pieperet et al. 2018 [36]	Y	Y	Y	Y	N	Y	Y						4 high
Rostad et al. 2018 [37]	Y	Y	Y	U	Y	Y	U						3 moderate

## Data Availability

Data is contained within the article or Supplementary Materials.

## Supplementary Materials

The following are available online at https://www.mdpi.com/article/10.3390/geriatrics6040101/s1. Table S1: Search Strategy. Table S2: Data Extraction Form. Table S3: Psychometric Evaluation of Pain Assessment Tools. Figure S1: Narrative Synthesis Model.
