# Supplementary material for: Pain Assessment for Individuals with Advanced Dementia in Care Homes: A Systematic Review
Source: Geriatrics (Basel). 2021 Oct 19;6(4):101. doi: 10.3390/geriatrics6040101 (PMC8544573; doi:10.3390/geriatrics6040101)

Figure S1: Narrative Synthesis Framework

Based on guidance produced by the Economic and Social Research Council (ESRC), [43] (p.49)

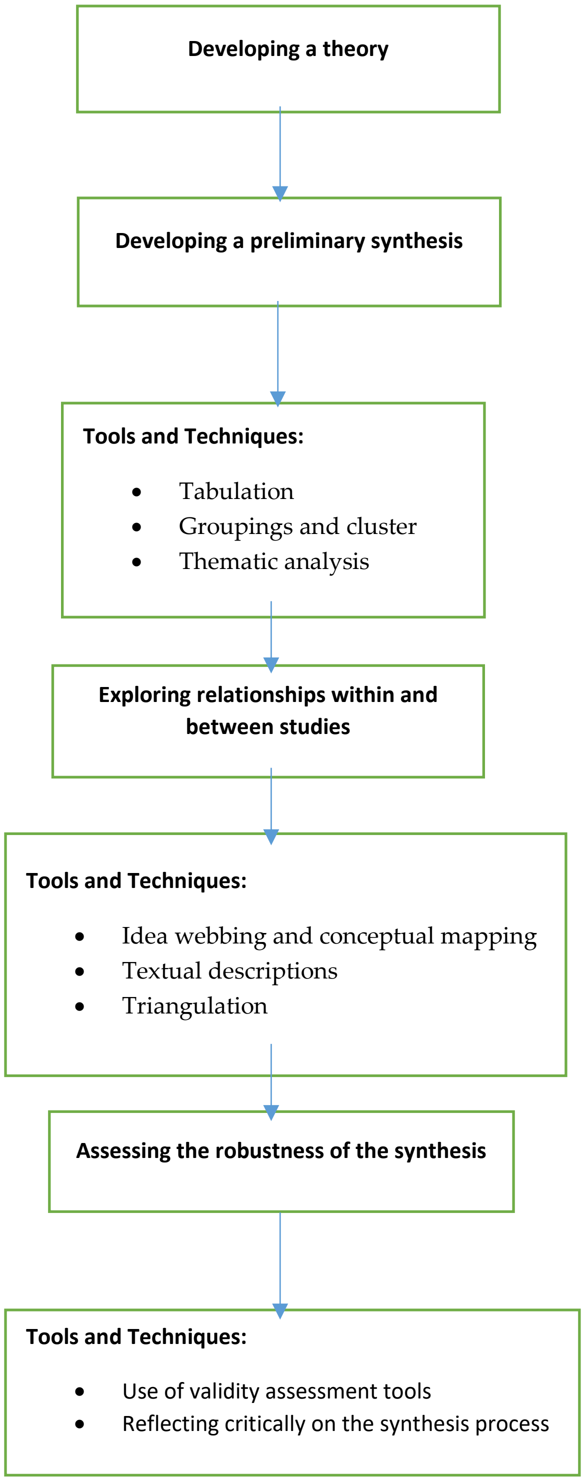

Supplement: Supplementary file 1 [file geriatrics-06-00101-s001.zip › geriatrics-1404136 supp/Figure S1; Narrative Synthesis Framework.pdf]
