# Supplementary material for: Pain Assessment for Individuals with Advanced Dementia in Care Homes: A Systematic Review
Source: Geriatrics (Basel). 2021 Oct 19;6(4):101. doi: 10.3390/geriatrics6040101 (PMC8544573; doi:10.3390/geriatrics6040101)
Supplement: Supplementary file 1 [file geriatrics-06-00101-s001.zip › geriatrics-1404136 supp/Table S1; Search Strategy.pdf]

Table S1: Search Strategy

Database(s): **Ovid MEDLINE(R)** 1946 to present
Search Strategy:

#	Searches	Results
1	*Dementia/	44306
2	*Dementia/di [Diagnosis]	6383
3	"Memory disorder*".ti,ab.	1796
4	"Cognit* impair*".ti,ab.	72959
5	"Lewy body".ti,ab.	4029
6	"Lewy body dement*".ti,ab.	964
7	"Fronto-temporal".ti,ab.	1903
8	"Fronto-temporal dement*".ti,ab.	303
9	"Dementia syndrome".ti,ab.	571
10	"Mental degenerat*".ti,ab.	12
11	Alzheimer*.ti,ab.	155937
12	"Alzheimer* disease".ti,ab.	140379
13	"Vascular dement*".ti,ab.	6769
14	"Mental* deterior*".ti,ab.	1097
15	"Mental* disorder".ti,ab.	9128
16	"Mental* derang*".ti,ab.	78
17	"Advanc* dement*".ti,ab.	1191
18	"Severe dement*".ti,ab.	1868
19	"Progress* dement*".ti,ab.	1795
20	"late* stag* dement*".ti,ab.	153
21	"clinical dementia rating".ti,ab.	2420
22	"Global deterioration scale".ti,ab.	461
23	"Moderate to late stage* dement*".ti,ab.	1
24	"End stage* dement*".ti,ab.	142
25	or/1-24	257776
26	"Retir* home*".ti,ab.	317
27	"care home*".ti,ab.	4401
28	"Residential home*".ti,ab.	1004
29	"Residential care home*".ti,ab.	382
30	"Residential care".ti,ab.	3557

31	"Long-term residential care".ti,ab.	177
32	"Long-term residential care home".ti,ab.	0
33	"Aged-care facilities".ti,ab.	997
34	"Nursing home*".ti,ab.	31373
35	"Nursing care home*".ti,ab.	87
36	"Long-term care home*".ti,ab.	343
37	"Convalescent home".ti,ab.	71
38	"Old people's home".ti,ab.	113
39	"Old folks home".ti,ab.	6
40	"Convalescent hospital".ti,ab.	64
41	"Retirement home".ti,ab.	154
42	"Rest home".ti,ab.	102
43	"Geriatric home".ti,ab.	98
44	Nursing Homes/	36810
45	Long-Term Care/	26950
46	exp Residential Facilities/	55223
47	Home Care Services/	34849
48	Intermediate Care Facilities/	711
49	Home Care Services, Hospital-Based/ or Home Health Nursing/	2327
50	or/26-49	123668
51	Pain.ti,ab.	661454
52	*Pain/di [Diagnosis]	4360
53	"Pain assessment".ti,ab.	5753
54	"Pain assess* tool*".ti,ab.	716
55	"Pain tool*".ti,ab.	146
56	"Pain question*".ti,ab.	3684
57	"Pain scale*".ti,ab.	7084
58	"Pain thermomet*".ti,ab.	38
59	"Pain check*".ti,ab.	60
60	"Pain measure*".ti,ab.	2687
61	*Pain Measurement/mt [Methods]	5932
62	*Pain Measurement/st [Standards]	846
63	Psychometrics/is [Instrumentation]	5862
64	"Pain evaluat*".ti,ab.	1463

65	"Pain gauge".ti,ab.	1
66	"Pain valuat*".ti,ab.	4
67	"Pain tolerance".ti,ab.	1644
68	"Visual Analogue Scale".ti,ab.	26246
69	"Geriatric pain measure*".ti,ab.	20
70	*Geriatric Assessment/mt [Methods]	5208
71	"Face Pain Scale".ti,ab.	28
72	PAINAD.ti,ab.	88
73	PainAd.ti,ab.	88
74	DOLOPLUS-2.ti,ab.	37
75	Doloplus-2.ti,ab.	37
76	PACSLAC.ti,ab.	39
77	NoPain.ti,ab.	5
78	NOPAIN.ti,ab.	5
79	DS-DAT.ti,ab.	24
80	DisDat.ti,ab.	3
81	"Bolton pain assessment tool".ti,ab.	1
82	"Pain assessment for the dementing elderly".ti,ab.	3
83	PADE.ti,ab.	363
84	Paine.ti,ab.	85
85	"Pain assessment in advanced dementia scale".ti,ab.	29
86	"Pain assessment checklist for seniors with limited ability to communicate".ti,ab.	27
87	CNPI.ti,ab.	50
88	"Pain assessment in advanced dementia".ti,ab.	91
89	PACSLAC-II.ti,ab.	7
90	"Elderly pain caring assessment 2".ti,ab.	1
91	EPCA-2.ti,ab.	14
92	Algoplus.ti,ab.	19
93	"Mobilization-Observation-Behaviour-Intensity-Dementia".ti,ab.	10
94	MOBID-2.ti,ab.	17
95	"Facial Pain Scale".ti,ab.	38
96	CPAT.ti,ab.	67
97	ECPA.ti,ab.	76
98	"Mahoney pain scale".ti,ab.	1

99	"Pain assessment scale for use with cognitively impaired adults".ti,ab.	1
100	REPOS.ti,ab.	108
101	FLACC.ti,ab.	358
102	or/51-101	683380
103	25 and 50 and 102	807
104	limit 103 to (english language and humans)	716
