# Supplementary material for: Pain Assessment for Individuals with Advanced Dementia in Care Homes: A Systematic Review
Source: Geriatrics (Basel). 2021 Oct 19;6(4):101. doi: 10.3390/geriatrics6040101 (PMC8544573; doi:10.3390/geriatrics6040101)
Supplement: Supplementary file 1 [file geriatrics-06-00101-s001.zip › geriatrics-1404136 supp/Table S2; Data Extraction Form.pdf]

Table S2: Data Extraction Form

Author, Date and Country	Aims	Condition	Sample/ description of population	Setting	Method	Assessment tool used	Main findings	Recommendations	Authors conclusion
Atee, M *et al* (2018) Australia	To examine the inter-rater reliability of the electronic Pain Assessment Tool (e-PAT) among raters when assessing pain in residents with moderate-severe dementia	Predominantly severe dementia (80%)	**Sample size 10** rated by 11 care staff (1 clinical nurse, 4 registered nurses, 5 enrolled nurses, 1 care worker). Age range of 63.1-84.4 years (mean 74.4 years), gender ratio of 50:50, 90% were Caucasians.	Dementia-specific residential aged care facility	2 week observational study using a convenience sampling technique. The study uses kappa statistics as an appropriate tool to measure inter-rater reliability. Staff who consented to participate attended an education and training session prior to the study, conducted by the PI. Staff were recruited if they had	ePAT	ePAT demonstrated good reliability properties in assessing pain in this population. Kappa statistics were excellent when assessing at rest and moderate when assessing during movement. However, a linear model confirmed agreement did not depend on conditions (rest/movement). Additionally,	Patients with dementia have an enhanced facial activity and observational tools improve pain recognition in this population, so ePAT is recommended to facilitate the process of pain detection in these patients. However, due to the small sample and short time frame, generalisation to other settings and populations are limited.	Combining automated facial expression analysis and clinical behavioural indicators in a single observational pain assessment scale affords ePAT good reliable properties. This supports its use with nonverbal residents with advanced dementia. However there is currently no gold standard

					been working for 3 months in the facility (experience ranged from 1 - 30 years), were familiar with the residents and were present for duration of the study. Testing was completed by 2 raters at a time who were blinded to each others assessment, which took place during routine nursing activities or activities of daily living, including rest and movement.		the face domain was highly correlated with gold standard measures of self-reporting on movement, supporting psychometric properties of ePAT. The face domain of ePAT has good sensitivity to the presence of pain.<br>One participant had only two pairs of ePAT ratings as opposed to four but the reason for this is unclear.	Additionally the authors are disclosed as shareholders in the ePAT instrument, indicating a risk of bias.	pain assessment tool for this population and any attempt to work towards this goals should be encouraged.

Author, Date and Country	Aims	Condition	Sample/ description of population	Setting	Method	Assessment tool used	Main findings	Recommendations	Authors conclusion
Cheung, G., Choi, P. (2008) New Zealand	A pilot study to evaluate the inter-rater reliability of the PACSLAC when administered by care staff	Predominantly severe dementia (30/50), screened using MMSE	Sample size 50 rated by 12 care givers. Two had been excluded as they did not require assistance during their personal care. The mean age was 89.9 years,72% female/28% male, mean MMSE score was 7.5 (this is a screening tool and used to contribute to diagnosis and progression of dementia)	Four specialist dementia rest homes in Hamilton and Cambridge in New Zealand	A pilot observational study, to evaluate the inter-carer reliability of the PACSLAC in assessing pain in severe dementia. Residents were observed and rated during their usual personal care. Care givers were given an hour in-service training on using PACSLAC. The 50 residents were classified into Group 1 (MMSE score <1) and Group	PACSLAC (Pain Assessment Checklist for Seniors with Limited Ability to Communicate)	The study found that residents with more severe dementia have higher PACSLAC scores. Pearson correlations were strongly significant which supports inter-rater reliability of PACSLAC. However ratings were taken by medical undergraduate with little experience in dementia care, to compare to those by	Using a standardised pain assessment tool increases caregivers awareness and encourage them to take the process of pain management more proactively. Caregivers could be empowered as part of the treatment team. Also, inter-rater reliability when using PACSLAC can be improved with standardised training. This study has limited external validity as recruitment was	Using a standardised pain assessment tool can improve pain management of this group, improve their quality of life and empower care givers. Further research is needed involving different care centres with standardised training provided by qualified trainers.

					2 (MMSE score >10)		experienced caregivers.	in one part of New Zealand.	
**Author, Date and Country**	**Aims**	**Condition**	**Sample/ description of population**	**Setting**	**Method**	**Assessment tool used**	**Main findings**	**Recommendations**	**Authors conclusion**
Cohen-Mansfield, J. (2006) USA	To describe the validation of an informant-based pain assessment for persons suffering from dementia called Pain Assessment in Noncommunicative Elderly Persons (PAINE)	Dementia, the majority having severe dementia	The first study had a **sample size of 80** residents of a nursing home where they had resided for an average of 3.45 years. The average age was 87 years, a majority being 84% female. 49 of these participants were classified as having severe dementia. The second study had a **sample size of**	Nursing homes	Two different samples were used to demonstrate reliability and validity of the PAINE assessment tool for pain in individuals with severe dementia. Internal consistency was explored in sample 1 using Cronbach and test-retest reliability using Pearson correlations, with validity tested using receiver	Pain Assessment in Noncommunicative Elderly Persons (PAINE). Study 1 also used a geriatrician physical assessment and a relatives assessment by questionnaire. Study 2 compared PAINE to other observational assessment	The results show adequate internal consistency of PAINE and both interrater and test-retest reliability. PAINE also shows reasonable correlations with existing measures of pain in people with dementia. PAINE has the advantage of using a comprehensive list of pain symptoms on the basis of systematic questioning of	There is a growing set of pain assessments for persons with dementia, the general categories being self-report (for earlier stages of dementia), direct observation and informant ratings. Clinical practice should utilise a combination of methods due to the complexity of the task. Informant based tools, such as PAINE, are intended to be	The validity results suggest that PAINE could be a useful tool in detecting pain in this population. Future studies should illuminate the practical utility of observational assessments in this population, as they can be time consuming and require training.

			**91** from 2 nursing homes where they had resided for an average of 3.3 years. The average age was 89 years, 84% being female.		operating characteristic curve analysis. In the second sample validity was tested by correlating scores on PAINE with those from other assessments.	PAINAD and CNPI. Trained research assistants then administere d a questionnai re to nursing staff who had recorded the observation .	caregivers from different institutions. This contributes to external validity of the results, although there is a lack in supporting data presented; for example there are no direct quotes or themes identified from the questionnaires	used by carers who are most likely to observe the pain and can use a standardised method to facilitate detection of pain.	
**Author, Date and Country**	**Aims**	**Condition**	**Sample/ description of population**	**Setting**	**Method**	**Assessment tool used**	**Main findings**	**Recommendatio ns**	**Authors conclusion**
Cohen-Mansfield *et al* (2007) USA	To examine the utility of nine pain assessments in identifying pain and documenting	Advanced dementia	**Sample size 121** nursing home residents (out of which there were 63 classified	Nursing homes	Open medication study over a number of phases (up to 8 weeks); after 2 weeks of	Observation al pain assessment used were CNPI, PAINAD and OPBAI.	The currently available assessments can identify those persons who could be	The findings suggest that utilising multiple assessments can be beneficial in detecting pain that will respond	Those receiving treatment showed reduced levels of pain, which should

	responsiveness to pain medication in nursing home residents with dementia		as being in pain), mean age 88, primarily female (81.8%). Diagnosed with dementia and having resided in the facility for at least 2 months. Exclusions included those without dementia, a high MMSE, or on narcotics.		medication in each phase pain assessments were obtained in the same manner as baseline. If they were still in moderate pain then the attending physician ordered the next phase of medication. Each phase was 2 weeks to enable staff to have sufficient time to notice changes in behaviour. Participants were involved for up to four phases, determind by individual responses to	Those evaluated as having pain were included in the pain medication protocol.	helped with analgesia. Three comparison groups were utilised to help separate the effect of intervention from random fluctuations in pain perceptions and the passage of time. Intraclass correlation coefficient, to assess agreement rates, was 92-94%. A two way repeated measures ANOVA calculated the F-value for each instrument, comparing	to pain medication. Also, the findings promote a much wider use of analgesics in this population in order to reduce symptoms. Behavioural signals observed by carers may provide better indicators for the need to treat pain than self-report (gold standard) in those with advanced dementia	promote a much wider use of analgesics in this population. Education of family and physician is also necessary in providing care to this population, with alleviating pain impacting on their quality of life.

					pain medication. Participants were blind to the treatment, as were the research assistants.		pain levels of those in different groups. This showed a significantly greater decrease in pain in the persons who received the pain protocol. PAINE and PADE were the strongest detecting treatment effects.		
**Author, Date and Country**	**Aims**	**Condition**	**Sample/ description of population**	**Setting**	**Method**	**Assessment tool used**	**Main findings**	**Recommendations**	**Authors conclusion**
Ersek, M (2020) USA	To examine the frequency and severity of pain and use of pain therapies among long-term care residents	Moderate to severe dementia (87% and 81% severe dementia in each group)	**Sample size 205**, residing at the care facility for at least 7 days. Average age of 84 years, 47% female,	16 long-term care facilities in Alabama, Georgia, Pennsylvania, and	A comprehensive pain assessment protocol was used to evaluate pain	Expert Clinician Pain Intensity Rating (ECPIR), Nursing Home Staff	Most pain was found to be intermittent and moderate to severe in intensity. Differences between	Direct observational tools should be augmented with a comprehensive, interdisciplinary	Using a comprehensive pain assessment protocol, it was found that most nursing

	with dementia and to explore the factors associated with increased pain severity.		69% white, with an average of 2.5 painful conditions.	New Jersey	severity and characteristics through medical record review, interviews with nursing home staff, physical examinations, as well as pain observation tools. An initial sample of 105 was recruited without regard to pain status so to include absence of pain. The method then targeted residents who nursing staff reported had moderate to severe pain to balance the final sample	Pain Ratings, Mobilization - Observation -Behaviour-Intensity-Dementia Pain Scale (MOBID), Pain Intensity Measure for People with Dementia (PIMD)	moderate and severe cognitive impairment was not statistically significant. Compared to expert clinicians, staff nurses estimates of pain was generally lower. Of residents, 90% received a pain therapy, with acetaminophen (87%) and opioids (32%) commonly utilized. Only 3% had a nondrug therapy documented in	assessment of pain. Pain related behaviours are higher during movement so this should be included in observational assessments. Nondrug pain therapies are under utilised in this population, it may mean there is a lack of empirical evidence to support the effectiveness of such intervention in this group, or it may be that it is inappropriate or there is a lack of provision. Further research is required.	home residents with moderate to severe dementia had mild usual, intermittent pain and the vast majority received at least one pain therapy in the previous week. Although these findings reflect improvements in pain management compared with older studies, there is still room for improvement in that 45% of the sample experienced moderate to severe pain at some

					across pain levels. Using the final sample, a mean pain intensity score was determind for residents with moderate and severe cognitive impairment.		the medical record. Multivariate linear regressions are also used to account for variables, finding that opioids are significantly associated with pain severity.		point in the previous week
Author, Date and Country	Aims	Condition	Sample/ description of population	Setting	Method	Assessment tool used	Main findings	Recommendations	Authors conclusion
Fuchs-Lacelle, S *et al* (2008) Canada	To determine whether systematic pain assessment leads to improved pain management practices and reduces nursing stress	Severe dementia	**Sample size 181 (of which 101 completed the study)** in 21 units across 12 long-term care facilities. Participants were >65 years old, with severe	Long-term care units in a midsize metropolitan area. The units were either within a long-term rehabilitation	A 3-month comparative longitudinal design study. This involved an experimental group, in which the nursing staff regularly assessed pain using an	Pain Assessment Checklist for Seniors with Limited Ability to Communicate (PACSLAC). A number of other measures were used,	The main findings showed that pain scores, as measured by PACSLAC, showed a statistically significant difference between the groups. It found a reduction in the rate of nurses stress	The findings support the importance of routine systematic pain assessment in long-term care for individuals with severe dementia. It also supports the clinical utility of the PACSLAC tool, as this resulted in	It can be concluded that a systematic approach to assessment is beneficial to the individuals with severe dementia but also their care givers.

			dementia and severe communication impairments. Participants were identified by nursing staff. In the experimental group there was an average age of 84.89 years and 70% female. In the control group the average age was 85.39 and 88% female. Patient primary carergivers were invited to participate in the study of staff	hospital or long-term care home.	observational pain tool, and a control group, which were assessed with an attention-control measure to record observed activities of the participants. Additionally, nursing stress and burnout was measured as well as medication administration. The groups were from different units to avoid contamination using random assignment	including Nursing Stress Scale, Burnout Scale, and Present Functioning Questionnaire.	and work-related emotional exhaustion in the group using PACSLAC, together with an increase in PRN medication in this group. The findings hold strong ecologic validity; the research was conducted in the clinical setting and by care staff who could be using the pain assessment tool in practice. This was modelled over , with adjustments for potential confounding effects.	a reduction in carer stress and an increase in prescription of medication compared to a control group. The high drop out rate over this length of study highlights the need for further research to examine staff experiences with systematic pain assessment and the potential barriers to compliance.	

			stress, however there was a high drop out with only 32.8% completing the study. A linear mixed effects regression model is used to compare the data. This allows inclusion of participants with missing data.						
Author, Date and Country	Aims	Condition	Sample/ description of population	Setting	Method	Assessment tool used	Main findings	Recommendations	Authors conclusion
Griffioen C *et al* (2017) Norway	To investigate the prevalence of pain, frequency of opioid prescription, and factors associated	Advanced dementia	**Sample size 327** Residents aged >65, diagnosed with moderate-severe , with	18 Norwegian Nursing homes	Cross-sectional analysis using baseline data from a multicentre cluster randomised trial conducted	MOBID-2 to assess pain, Cohen-Mansfield Agitation Inventory (CMAI) to measure	Pain, the associated number of diagnoses associated with pain and affective symptoms	Clinically nursing home residents with advanced dementia need regular assessment of pain and analgesic use is	Despite the extensive prescribing of opioids the prevalence of pain in this population remains high.

	with strong opioid use in nursing homes		significant behavioural disturbances. Mean age was 85.7 and 74% were female. Residents lived in a nursing home for at least 4 weeks. Exclusion criteria included those with severe medical diseases or other neurological or psychiatric disorders.		over 9 months (October 2009 – June 2010). The objective of the original study was to determine whether treatment of pain can reduce agitation in people with moderate to severe dementia and behavioural disturbance. Two groups were compared; those with and without opioid prescription. Regression analysis was used with strong opioid prescription use as the	agitation. As this study uses baseline data from a previous study, details of assessment methods were not present (such as who assessed, whether they knew the residents, whether they had been trained and under what circumstances)	(depression and anxiety) was independently associated with the prescription of strong opioids in nursing homes in residents with advanced dementia. There was a high pain rate despite opioid prescription, questioning the appropriateness of the assessment of pain. In the regression analysis nine variables were significantly associated with opioid	also regularly assessed. The study also suggests the results may indicate inadequate nonpharmalogical pain management as cotreatments. Further research is needed to explore this, together with further studies to explore the rationale for prescribing opioids.	This suggests that many nursing home residents with pain are inadequately treated

					dependent variable.		use. These were in the behavioural domain, medical domain or pain domain.		
**Author, Date and Country**	**Aims**	**Condition**	**Sample/ description of population**	**Setting**	**Method**	**Assessment tool used**	**Main findings**	**Recommendations**	**Authors conclusion**
Hadjistavropoulos *et al* (2018) Canada	To compare a Facial Action Coding System (FACS) based fine grained system to the PACSLAC-2 in differentiating painful from non-painful states in older adults with and without dementia	Moderate-Severe dementia with severe limitations in ability to community. A second community group without dementia as a comparison.	**Sample size 48 + 52 = total number 100.** Dementia group n=48: average age 82.5, female 36, male 13, years of education 13.5, CPS score 3.74 (moderate-severe) with severe limitations in ability to communicate as identified	Long-term care facilities within a mid-sized metropolitan area	Observational study. Residents with dementia and older adults without dementia were video-recorded during a quiet baseline condition for 5min and while they took part in a physiotherapy examination designed to identify painful areas. Videos were	FACS and PACSLAC-2	The results support the use of non-verbal pain assessment tools in community dwelling seniors and seniors with limited ability to communicate due to dementia. However there may not be clinical advantage in conducting	PACSLAC-II can be used in a clinical situation involving a standardised protocol of movements. This standardised approach assists with clinical comparisons and evaluations. Further research is needed to study the value of fine-grained facial responses, as evaluated by FACS. It would	Clinically PACSLAC-2, or other similar scales, are valuable and valid tools in practice and there may not be any clinical advantage in using a resource-intensive coding approach based on FACS.

			by health care staff. Community sample n=52; average age 75.46, female 33, male 23, years of education 12.51.		randomised and coded using pain-related behaviours from FACS and PACSLAC-2. Trained coders completed the coding frame by frame to ensure rigour. An independent coder watched the videos and completed the PACSLAC-II the way it would be performed in a clinical setting. Mixed model analysis of variance (ANOVA) was used to determine any group differences.		fine-grained behaviour coding compared to using an easy to use observation scale such as PACSLAC-2, although the facial behaviour items of PACSLAC-2 account for the most variance – the facial response to pain has more communicative value than other non-verbal responses. Participants with dementia scored higher on the PACSLAC-2 than those	also be valuable to cross-validate the PACSLAC-2 and the FACS in situations involving comparisons of painful versus non-painful movements.	

							without dementia. It supports other studies that demonstrate people with dementia tend to display increased non-verbal pain behaviours.		
**Author, Date and Country**	**Aims**	**Condition**	**Sample/ description of population**	**Setting**	**Method**	**Assessment tool used**	**Main findings**	**Recommendatio ns**	**Authors conclusion**
Hoti, K *et al* (2018) Australia	To examine the clinometric properties of the electronic Pain Assessment Tool (e-PAT) in dementia patients	79.4% of participants had severe dementia	**Sample size of 34** residents aged 68 – 93.2 years (mean 85.5) were included, 20 females and 14 males. 35.3% had Alzheimer's dementia and 44.1% had an unspecified type of	Residential aged-care facilities	Observational study using purposive convenience strategy, data being extracted from a previous observational study of ePAT over a 10 week period. Pain assessments were done twice (at rest and post	ePAT, which uses smart device-enabled app with automated facial analysis in conjunction with clinical indicators, compared to the Abbey Pain Scale.	The ePAT has strong clinimetric properties, providing evidence of how it can be used clinically in relation to pain detection in the target population	This suggests the use of the ePAT in the context of assessment and management of pain in patients with moderate-severe dementia can result in a reduction in under-recognition and under-treatment of their pain. The	The findings reveal strong clinimetric properties of the ePAT. Its predictive validity and CU are excellent, giving further evidence to the quality of the pain assessment attained by ePAT. Clinical utility is

			dementia. All were Caucasian except for 1.		movement) in pairs of raters who were blinded to one another's assessments and to pain therapies received by the residents. Statistical analysis was methodical and covered a number of variables. For example the Mitchell's Index was used to investigate the clinical utility of the ePAT tool, taking into account variables such as type of dementia and different pain conditions.			ePAT is the only pain assessment tool that uses automation and so would have positive effects on pain detection (and communication of this to physicians) for a group whose ability to report pain is compromised.	important in assisting with the decision-making process about pain management

Author, Date and Country	Aims	Condition	Sample/ description of population	Setting	Method	Assessment tool used	Main findings	Recommendations	Authors conclusion
Husebo, B *et al* (2009) Norway	To examine the intra- and inter-rater reliability of pain behaviour indicators, inferred pain intensity and the overall MOBID pain score	Severe dementia	**Sample size of 26** participants with a mean age of 87 years, 89% were female and 81% were widowed. They had lived in the care home for a mean of 34 months, and severe dementia was measured using the MMSE. All had a pain diagnosis, and all but one patient received one or more	The largest Nursing Home in Norway	Observational study during video uptake, observed by two groups; primary caregivers who knew the residents and LPNs who did not know the residents. All received the same 2 hour briefing on dementia, behaviour, assessment of pain and use of the tool. Each film sequence lasted 7-10minutes, resulting in 260min of video material. Rating of all	MOBID Pain Scale	MOBID was found to have moderate to excellent intra- and inter-rater reliability of pain intensity for each item. When investigating pain behaviour indicators, varying degrees of reliability were found. The intensity of pain behaviours rather than the number could be considered important when entering the process of pain treatment.	Reliability of the MOBID pain scale in ordinary clinical practice remains to be examined. It is speculated whether scoring by video uptake over-estimates pain observations or whether hands on situations underestimate them, further research is required to include larger samples and different settings. It is also recommended that, due to the challenge to capture pain	The observation of pain behaviours may help with the assessment of pain intensity, even though reliable categorisation of pain behaviours was not a prerequisite for scoring pain intensity reliably. Interpretation of the overall pain intensity depends on observations of pain behaviours as well as the

			analgesic (19% opioid). Recruitment was by purposive sampling according to the strict inclusion/ exclusion criteria in order to address the research question. Participants had to have severe dementia, be over 65 years and have an advocate. Exclusion criteria included delirium, psychosis or on short stay or palliative care.		videos was performed within one day. LPNs could not have contact with each other during this time. Movements were standardised and guided by primary care givers. Intra- and inter-rater reliability was measured by intraclass correlation coefficient.			behaviours in this population, particularly when the observers do not know the patient – so this is an important variant in assessment. The care givers interpretation of what pain means to the patient is important; to interpret pain behaviours and the transformation to pain intensity. This highlights the value of systematic observations of pain behaviour during standardised activities.	whole care situation.

Author, Date and Country	Aims	Condition	Sample/ description of population	Setting	Method	Assessment tool used	Main findings	Recommendations	Authors conclusion
Husebo, B *et al* (2014) Norway	To explore MOBID-2 test-retest reliability, measurement error and responsiveness to change. The study also had other hypotheses, including the association between pain and neuropsychotic symptoms and activities of daily living	Severe dementia	**Sample size 352**, with advanced dementia. At baseline the characteristics between the control and intervention groups were similar. Most were women (74% and 78% respectively), mean age of 87 and 85. There is no consideration of socio-economic or ethnic status. The recruitment strategy is described	Nursing homes – 18 home through 5 municipalities in Western Norway	Analyses was based on data from a **Cluster RCT** over an 8 week period. The original RCT was studying behavioural disturbances, measuring agitation, in conjunction with treating pain. Primary carergivers were assessors, who knew the resident for at least 4 weeks. They all had a 2 hour education programme. There was a randomised	MOBID-2, CMAI (agitation), FAST (ADLs)	The standard error of measurement and the smallest detectable change in connection with the MOBID-2 indicate that it is responsive to a decrease in pain after a stepwise protocol for treatment in pain. Test-retest reliability was demonstrated as satisfactory, are clinically relevant and beyond measurement error.	MOBID-2 can be used clinically to improve pain treatment decisions	Indications were provided that MOBID-2 pain scale is responsive to a decrease in pain intensity over time, after pain treatment for individuals with advanced dementia.

			elsewhere, but assignment to groups was randomised and assessors were blinded to group allocation.		control group (individuals not expected to change, determind by using intraclass correlation coefficient)		All patients were accounted for and analysed, it included 8 deaths in the control group and 6 in the intervention group.		
**Author, Date and Country**	**Aims**	**Condition**	**Sample/ description of population**	**Setting**	**Method**	**Assessment tool used**	**Main findings**	**Recommendatio ns**	**Authors conclusion**
Jordan, A et al (2011) UK	To investigate the utility of PAINAD in assessment of pain in individuals with advanced dementia	Advanced dementia (using Clinical Dementia Rating CDR)	**Sample size 79;** 72% female, 32% male, mean age 82, mean time in home 36 months. Type of dementia recorded, ethnicity mentioned but not presented. Participants were selected by	Nursing homes; 1 NHS continuing care unit for severe dementia and 3 private EMI homes. North Tyneside	Observational study over three months, conducting three observed occasions (rest, mealtime and time of intervention) by researcher or nurse to determine whether the participants were in pain using PAINAD. Each observer	PAINAD (and DisDAT)	PAINAD has 92% sensitivity, suggesting that if a person with severe dementia is in pain this tool is likely to detect it. However there were high levels of false positives (33%), so other potential causes of behavioural	PAINAD and other screening tools should be used as part of a fuller assessment in the detection of pain in people with severe dementia	PAINAD is a sensitive tool for detecting pain in this population but frequently detects psycho-social distress rather than pain. It can also be used to detect when pain management strategies have been successful

			purposive sampling in order to identify the target population with strict inclusion criteria.		was blinded to the result recorded by the other observer. The cause of any observed pain was then discussed with nursing staff. Those who were found not to be in pain formed the false positive group. All were reassessed at one and three months. Changes in scores using PAINAD were analysed using the Wilcoxon signed ranks test and differences between scores for each		should be explored.		

					group were analysed using the Kruskal-Wallis and Mann-Whitney tests.				
**Author, Date and Country**	**Aims**	**Condition**	**Sample/ description of population**	**Setting**	**Method**	**Assessment tool used**	**Main findings**	**Recommendations**	**Authors conclusion**
Jordan, A *et al* (2011) UK	To evaluate the utility of a distress tool and a pain tool, considering the complex relationship between pain and distress and the multitude of causes of distress	Advanced dementia	**Sample size 79** , 72% female, mean age of 82. The mean length of time since diagnosis was 71 months, and they had been residents for a mean 36 months.	Nursing homes; 1 NHS continuing care unit for severe dementia and 3 private EMI homes. North Tyneside	Observational study over 3 months; participants were observed on three occasions for approx. 5min at a time of rest, mealtime and a time of intervention. Both tools were used, randomly assigned to a researcher or a nurse. Training was provided.	PAINAD and DisDAT	PAINAD has high sensitivity (92%) to pain but low specificity (62%). DisDAT can be used to identify pain caused by distress, encouraging broader thinking about distress in dementia. There was a significant improvement in both the PAINAD and DisDAT scores	Both tools could be used to pick up pain and to assess effectiveness of treatment, but have their limitations. The pain tool can also pick up distress not caused by pain. Distress is a major component of behavioural and psychological symptoms of dementia, and DisDAT could play an	Both tools are useful but the pain tool can also pick up distress, leading to false ascriptions of pain. The distress tool picks up a broader array of signs, which may be useful both in practice and in research.

							one month after a change in management.	important role in identifying and managing distress in this population with a rational approach to understanding the underlying causes.	
**Author, Date and Country**	**Aims**	**Condition**	**Sample/ description of population**	**Setting**	**Method**	**Assessment tool used**	**Main findings**	**Recommendations**	**Authors conclusion**
Lints-Martindale A, *et al* (2012) Canada	To investigate six observational pain assessment measures under two different pain conditions	Residents with a diagnosis of dementia, and the average MMSE score of participant was found to be 5.35 indicating severe dementia	**A sample of 136** identified with the assistance of nursing staff, but 12 died before completion of the study so leaving a **sample of 124** (88 women and 36 men) with a mean age of 83.94. The majority were	Long-term care facilities	Observational study. Participants were filmed during pain conditions (baseline, during vaccination and during movement-exacerbated pain which were identified by nurses familiar with the individual).	Self-reported Measure of Pain (CAS), although this could not be used due to level of impairment. ADD, CNPI, PACSLAC, PADE, PAINAD, NOPAIN	All of the tools used had adequate psychometric properties and were able to differentiate between non-painful and painful states, even after controlling for the different duration of each pain condition. There were	Behavioural measures tap into different dimensions of the pain experience, and behavioural observations are less susceptible to confounding variables (such as demand and situational characteristics) and should be most important for pain	The findings provide much needed psychometric information for pain assessment tools. They also support the utility of the comprehensive coverage of the AGS recommended behavioural domains of

			widowed (n=65) or married (n=39). An average of 10.63 years of education and a mean of 5.39 of medical diagnosis		The films were placed into a randomised viewing order and coded with the selection of pain measures by a research assistant. Cronbach's alpha calculated internal consistency, Cohen's Kappa correlation were calculated to assess inter-rater reliability and ANOVA used to test variables covariates such as differences between vaccination and movement-		high levels of inter-rater agreement. Internal consistency was highest for PACSLAC and PAINAD. All six observational pain measures were significantly correlated with each other.	assessment in this population. The results recommend use of the AGS behavioural domains found in PACSLAC without adding administration burden.	pain assessment. Additionally, until there is a standardisation of pain assessment protocols, an individualised approach is recommended.

					exacerbated pain.				
**Author, Date and Country**	**Aims**	**Condition**	**Sample/ description of population**	**Setting**	**Method**	**Assessment tool used**	**Main findings**	**Recommendations**	**Authors conclusion**
Mahoney, A *et al* (2008) Australia	To describe the development and trial of the Mahoney Pain Scale which aims to assess pain in advanced dementia and distinguish it from agitation. There is no standard method for differentiating pain from agitation.	Advanced dementia	135 participants recruited of which 18 died, leaving a **sample size of 112** (87 women, 32 men). Mean age 85.4, with a mean of 3.8 years spent in the home. All participants had a diagnosis of advanced dementia. The primary author systematically allocated into 1 of 4 groups (null	16 nursing homes across New South Wales	30 nurses completed use of the MPS 28 RNs and 2 NAs). The MPS is administered by caregivers who know the person well. The primary author conducted 2 hour training sessions. Interrater reliability assessed by blind assessment of four groups, with two nurses independently administering the MPS.	Mahoney Pain Scale (MPS)	Interrater reliability was acceptable overall. Inter-rater agreement was stronger during the aversive conditions, so making it easier to concur pain signs.<br>92% of nurses agreed MPS was not hard to use and that it gives an accurate assessment of pain.<br>76% agreed that the MPS raises their	Nurses responses support the clinical feasibility of MPS.<br>The MPS may be useful for assessing pain in advanced dementia as can differentiate between pain levels, agitation or neither. However further research is needed, using larger samples to understand pain presentations in this complex group.	Results suggest that MPS is clinically relevant. It has provided initial evidence for the reliability, validity and clinical feasibility of the MPS. However there is risk of reporting bias. Development of the pain tool is closely associated with the primary author. More research is required to examine how to integrate

			group, pain group, agitation group, combined group) so may involve bias.		A Pain Scale Usage Questionnaire was completed for clinical feasibility.		awareness of their residents pain expressions		standardised pain assessments into clinical practice.
**Author, Date and Country**	**Aims**	**Condition**	**Sample/ description of population**	**Setting**	**Method**	**Assessment tool used**	**Main findings**	**Recommendations**	**Authors conclusion**
Malara, A (2016) Italy	To define the prevalence of pain in people with dementia in long term care facilities and to study the relationship between pain and Behavioural and Psychological Symptoms of Dementia (BPSD).	Diagnosis of dementia Severe Cognitive impairment 62%, moderate 27%, slight 11%	**233 sample size** (150 female and 83 males) diagnosed with dementia and chronic pain, mean female age 80.7 and mean male age 85.6. Participants were subjected to multidimensional and multidisciplinary	10 long-term nursing care facilities associated with the ANASTE (National Association of Third Age Residences) in Calabria, Italy	Prospective observational and descriptive study. The study was conducted by carrying out the customary care practice normally provided to all patients. Statistical analysis was elaborated by qualitative and quantitative variables. The logistic	Self-reporting NRS, PAINAD, Cornell and Cohen-Mansfield Agitation Scale	There is a close relationship between pain and behavioural and psychological symptoms in patients with dementia, in particularly with anxiety, irritability and aggression. Pain is often communicated in behaviour. There is also a significant	Pain should be assessed in a multi-dimensional context in patients with dementia in long-term care. Further research is needed to clarify the exact patho-physiological mechanisms underlying behaviour and pain.	Instruments of self-report alone are not sufficient to assess pain in people with dementia, especially in the advanced stages. Observational tools for pain should be used in a multi-dimensional assessment of pain.

			evaluation. 77.7% of the sample met the criteria for dementia.		regression model analysed statistical significance between pain, depression, irritability, anxiety and drugs.		correlation between pain and depression, implying these conditions often coexist.		
**Author, Date and Country**	**Aims**	**Condition**	**Sample/ description of population**	**Setting**	**Method**	**Assessment tool used**	**Main findings**	**Recommendations**	**Authors conclusion**
Monacelli, F *et al* (2013) Italy	To evaluate use of the Doloplus-2 assessment tool in a group of the elderly with dementia to address analgesia	Moderate to severe dementia	**23 residents** selected by purposive sampling based on inclusion criteria; aged > 75 years, persistent pain, dementia diagnosis. Exclusion criteria; psychomotor agitation.	1x nursing home in Italy	Observational study for one year. An experienced nurse working in the home and trained in using the tool administered the scale with a monthly time schedule, taking approx. 8-10 minutes. It is unclear how many nurses took	Doloplus-2	The results showed a statistically significant reduction of the Doloplus-2 scale score between the first assessment and one year after of follow-up. In addition, collection of professional comments defined the	Due to the level of under-reporting and under-treatment of pain in this population, the regular use of a pain diagnostic tool is recommended, together with staff training and education. Regular administering of a pain	The study supports the utility of Doloplus-2 as a diagnostic tool for pain detection in this population. Impaired cognitive patients are less likely to receive analgesia resulting in cofounding

			18 were female, mean age 88.1, MMSE score rated advanced dementia. All participants contributed to the outcome measures		part and their standing. During observation all the patients attended usual activities of daily living including physiotherapy sessions. Statistical significance was calculated using Wilkoxon rank test and table of contingency when needed. A chief physician was responsible for analgesia prescription.		tool as handy and easy for clinical application (although no data or methods of this is provided, so reducing credibility). The data supports other literature for a systematic pain assessment in long term care, and increases staff awareness and attention to the importance of pain assessment in this population.	observation tool in nursing homes positively impacts on pain management in the care of dementia residents. However, this is a very small study with no control group so external validity is limited, although the longitudinal aspect of the study allows some preliminary conclusions.	factors (such as changes in behaviour) as a result of pain.

**Author, Date and Country**	**Aims**	**Condition**	**Sample/ description of population**	**Setting**	**Method**	**Assessment tool used**	**Main findings**	**Recommendations**	**Authors conclusion**
Neville *et al* (2014) Australia	To conduct a psychometric evaluation of three pain rating scales for people with moderate to severe dementia	Moderate to severe dementia (the majority with moderately severe to very severe)	**Sample size 126.** Mean age of 85.2, females 83, male 17. A dependent group of people for ADLs with a moderate level of chronic illness burden. Analgesics were the most commonly prescribed medication. Depression and BPSD moderate. 60% were	X4 RAFS (Residential Aged Care Facilities) in South-East Queensland, Australia	Observational study over four weeks (baseline, then x2 testing occasions two weeks apart). 26 nurses administered the pain rating scales and a questionnaire collected data about the nurses and the participants. The nurses received training before using the scales. Each participant were rated for pain at a	Abbey Pain Scale DOLOPLUS-2 and Checklist of Nonverbal Pain Indicators Scale (CNPI)	The findings provide evidence on the relative psychometric strengths of the three pain scales for people with moderate to severe dementia. Each of the scales showed medium correlations with the yes/no response initially completed by the nurses (this was to relate the	These scales should supplement clinical judgement and provide a standardised method to communicate and document pain. The participants were rated at rest with consideration of their experience of routine nursing care, but there are environmental aspects which cannot be controlled so open to	The lack of a suitable pain rating scale in clinical practice can mean inadequate information for maximising a person with dementia's well-being, or frustration for the nurses who are unable to effectively assess the pain.<br><br>The APS and the DOLOPLUS-2 are more likely to meet the need for the

			Australian born. Nurses – 58% were registered nurses and 33% were enrolled nurses and 9% nurses assistants		nominated time in mid-afternoon by two independent nurse raters (rater group 1 and rater group 2). Analgesia provided was consistent with usual practice. Demographic characteristics were analysed for continuous variables. Regression analysis was used to obtain an assessment of potential rater influences on pain scale scores. Four analysis were used to investigate		scales to the real world practice of nurses using their clinical judgement). The APS and DOLOPLUS-2 are better suited to measuring pain in this sample, both measures were well-supported by the factor analysis results. Less qualified nurse raters tended to under evaluate resident's pain.	confounding factors. Further research is needed in different settings and among people with different types of dementia to explore how this may impact on pain assessment.	benefit of nurses and people with moderate-advanced dementia in settings like RAFS.

					scale reliabilities, including Pearson and Cronbach's alpha coefficients. Two sets of factor analysis were conducted to investigate the construct validity of the three pain scales.				
**Author, Date and Country**	**Aims**	**Condition**	**Sample/ description of population**	**Setting**	**Method**	**Assessment tool used**	**Main findings**	**Recommendations**	**Authors conclusion**
Nowak, T (2018) Poland	The aim is to assess analgesia use among nursing home residents with moderate to severe cognitive impairment and to	Participants were screened to have at least moderate cognitive impairment	**96 residents**, over 65 (78 female), mean age 84, those with severe cognitive impairment on the Abbreviated Mental Test	2xNursing home facilities in the region of Wielkopolska in Poland	Observational study. All participants were assessed in activities of daily living, pain and agitation by trained nurses employed by the nursing	Abbey Pain Scale to assess pain, Cohen-Mansfield Agitation Inventory (CMAI) to assess agitation, Barthel	A relationship was found between the results obtained by the participants in the Abbey Pain Scale and the agitation scale. Importantly	Pain-related behavioural disturbances can be misinterpreted as symptoms of dementia, leading to inappropriate prescription of psychotropic	Pain can be an important underlying cause of behavioural disturbances in older subjects with dementia but the process of assessment is a

	delineate the relationship between pain and behavioural disturbance		Score (AMTS) was 82%. Analyzed subjects were very dependent on the Barthel Index. The mean number of drugs consumed was 5.5		home and who had knowledge of the resident. The Shapiro-Wilk was used to evaluate the normality of distribution of the variables. The Mann-Whitney test was used to investigate variables not normally distributed and Fishers test to assess between group differences. The correlation between pain and agitation was analysed using Spearman's rank correlation coefficient. Studied	Index to assess activities of daily living. Also, medicines were coded and analysed based on the classification system recommender by WHO.	subjects with a higher agitation (CMAI) received sedative drugs more frequently. They had a higher APS but did not receive analgesics more often than those with a lower CMAI. The study, however, did not take account of other potential causes of disruptive behaviours (such as depression, delirium) so further research is needed.	medication which are associated with increased risk of falls and death. It is therefore recommended that pain is routinely assessed and considered, with appropriate prescription of analgesia in this population.	challenging one. A stepwise, systematic approach to pain management in this group effectively reduces agitation.

					subjects were divided into two groups according to CMAI.				
**Author, Date and Country**	**Aims**	**Condition**	**Sample/ description of population**	**Setting**	**Method**	**Assessment tool used**	**Main findings**	**Recommendations**	**Authors conclusion**
Pieper *et al* (2018) The Netherlands	To assess whether implementation of the stepwise multidisciplinary intervention reduces pain and improves pain management	Advanced dementia (GDS of below 5)	**Sample size 288 residents in 21 clusters.** Eligible residents had advanced dementia, no other chronic psychiatric diagnosis, and clinically significant symptoms of pain and/or challenging behaviour. The mean age in the control group was 83.3, with 100	12 Dutch nursing homes with psychogeriatric units (mostly dementia) from the academic nursing home network.	Cluster RCT over 6 months. The nursing homes were randomised into a control group or an intervention group which involved implementation of the STA-OP protocol. The control group staff also received training, but not using the stepwise approach. The trial was single	PACSLAC-D (observation of pain) and MDS-RAI (estimation of pain)	Implementation of the stepwise STA OP intervention was effective in decreasing pain in nursing home residents with advanced dementia. The tailored stepwise and MDT approach was more effective. It was also found that behaviour cues for pain in dementia are	This protocol could be used effectively in other settings to manage pain in this population as it was possible to implement a tailored intervention trial within daily routines. Future research should examine more favourable implementation strategies, as there were questions about the fidelity of	This study indicates that STA OP can help healthcare professionals to realise effective pain management in addition to addressing behaviour in people with advanced dementia, which is crucial to an optimal quality of life.

			being female and 40 being male. In the intervention group the mean age was 84.3, with 107 being female and 41 male. 59 residents were lost during the study (29 in the control group and 30 in the intervention group) due to death or moving home.		blinded and units were allocated using a computer-generated sequence program. Pain was assessed in a standardised way by staff familiar with the individual, although they were not blinded so there was risk of the Hawthorne effect. Research assistants assessed all outcome variables. Differences in baseline characteristics were tested with the		essential for better pain management. Residents in the intervention group were less severely impaired, but both groups had a majority in severely advanced stages of dementia.	the knowledge, time and resources requirements required for such a multicomponent intervention in this design method.	

					Mann-Whitney U tests and ANOVA. Logistic GEE analysed dichotomous outcomes.				
**Author, Date and Country**	**Aims**	**Condition**	**Sample/ description of population**	**Setting**	**Method**	**Assessment tool used**	**Main findings**	**Recommendations**	**Authors conclusion**
Rostad *et al* (2018) Norway	The aim was to assess whether regular pain assessment using a pain assessment tool is associated with changes in pain scores and analgesic use in nursing home residents with severe dementia	Severe dementia	**Sample size 112.** A two step recruitment process was used with eligibility criteria being those who were over 65, with dementia who lacked the capacity for self-reporting pain (this was assessed by nurses and by the	16 nursing homes in four counties in Norway, each home being a cluster which were randomly allocated by an independent statistician using computer generation	Cluster RCT, 12 week intervention period. The experiential group was regularly assessed for pain using the Doloplus-2 twice a week and the control group received usual care. Random allocation of clusters (a home being a cluster) was carried out by	Doloplus-2	No overall effect of regular pain assessment was found on pain score or analgesia. The mean score of Doloplus-2 remained unchanged in both groups, with no overall effect found on pain prevalence tested with logistic regression for repeated	A standaradised, systematic approach to pain assessment may not be appropriate in advanced dementia care, but this should rather compliment clinical judgement and experience of staff, including their knowledge of the individual. Further research is needed on how	Further research is needed to examine a multi-method approach to pain assessment, and over a longer duration.

			investigator) Mean age was 83.6, the majority were female (69.9%). **15 participants were lost** due to death or moving (5 in the control group and 7 in the experimental) but no statistically significant differences were revealed between those who were lost and those who completed.	. 97 homes were invited, so there was a high level of declines/non-responses.	an independent statistician. Invitation by the computer-generated sequence took place between November 2014 and January 2016. The nursing homes, residents and next of kin were blinded to allocation and told the two groups were receiving different study procedures rather than different interventions. Mann-Whitney-Wilcoxan tests were used for continuous		measure. This does not support use of this pain tool, as did not lead to actions or decisions. It also suggests that further staff training may be required, as behaviour change could have been misinterpreted as a symptom of dementia rather than a sign of pain. The staff experience and knowledge of pain assessment varied.	standardised pain assessment can be used to support effective pain management in this population.	

					data. Linear mixed models were used to account for variables. Logistic regression was used to model pain prevalence as the dependent variable.				
Author, Date and Country	Aims	Condition	Sample/ description of population	Setting	Method	Assessment tool used	Main findings	Recommendations	Authors conclusion
Van Dalen-Kok (2019) The Netherlands	To study the application of PAIC to provide evidence of psychometric properties	Dementia, 71% in advanced stages	**Sample size 45** who met the inclusion criteria; a clinical diagnosis of dementia (of mild to moderate, although participant characteristic were predominantly severe	5 nursing homes within the university nursing home network in South Holland. Residents were selected by nursing staff from psychogeri	Multicentre observational study where residents were observed by nurses or nurse assistants during rest and movement. Assessors were provided with training by 30min video on how to use	Pain Assessment in Impaired Cognition (PAIC)	An observer agreement of >70% was found for most items of the body movement domain and vocalisation domain (based on American Geriatric Society domains). Pain was prevalent	Education and training might increase the clinical utility and feasibility of the PAIC. Further training of nursing home staff on how to use a pain measurement instrument to help identify pain in this group which	The pain assessment in dementia shows promising inter and intra observer agreement in a clinical setting. The PAIC needs further refinement in the clinical setting before it can be

			stage). Participants had a mean age of 85.7, predominantly female 80% and the average length of stay in the nursing home was 29.5 months.	atric wards.	the PAIC. Each resident was observed for 5-10min by four different, independent observers (two on two different days). Inter-observer and intra-observer agreement was assessed by percentage of agreement. Differences were analysed using McNemar's test for dichotomous variables and a Likert test was used to indicate the degree of presence of the 36 different		during movement but pain was also found during rest, which may suggest causes such as orofacial pain or neuropathological changes in the brain. Agreement was found to be lower in facial items, so this may indicate it is more difficult to assess or evaluate in the clinical setting.	impacts on quality of life. Further psychometric studies are required from other countries and cultures, with further development and refinement of this assessment tool.	recommended as a pain assessment tool for this population.

					items/indications of pain.				
**Author, Date and Country**	**Aims**	**Condition**	**Sample/ description of population**	**Setting**	**Method**	**Assessment tool used**	**Main findings**	**Recommendations**	**Authors conclusion**
Van Kooten (2017) The Netherlands	The aim of the study was to determine and compare pain prevalence, pain type and its pharmacological treatment in nursing home residents in relation to dementia subtype and dementia severity. It was part of the PAINdemiA study	Dementia, primarily severe	**The sample size was 199** The participants were over 60 with a diagnosis of dementia. They were mostly female (77.4%), mean age 84.9. The predominant dementia was AD (53.3%), primarily severe dementia (49.7% GDS 6 and 19.1% GDS 7)	10 Dutch nursing homes, in the dementia special care units	Observational cross-sectional study as part of the PAINdemiA study. All measurements were administered within a time frame of one week, over a period of 18months. The residents understanding of self-reporting scales and ability to communicate was judged by the researcher, so there could	MOBID-2 and self-report, PAINAD used in residents with difficulties to communicate their pain as judged by the researcher	Most residents did not show pain behaviour. But residents with more severe dementia experienced pain more often and with higher pain intensities than those with less severe dementia (27%v.15%). It also found that depressive symptoms complicate pain management. The study has a low response	Attention should be pain to the coexistence of depressive symptoms when assessing and treating pain. Regularly scheduled analgesics do not necessarily reflect optimal pain treatment. There was lower rates of opioid use, so increasing the dosages of analgesic drugs can significantly improve pain management. Systematic medication	Pain was observed more often in residents with severe dementia, so more focus should be on how pain management could use more tailored approaches and be regularly adjusted to individual needs.

					be an element of bias. Pain was observed during daily activities and guided movement during morning care by nursing staff. In all residents with an indication of pain, the type of pain was determined by the findings of the examination and the medical history. Descriptive statistics for continuous and non-normally distributed level variables		rate (32.2%) so limits the external validity. A majority of the sample were tested using self-reporting, despite the prevalence of severe dementia. This questions the validity of the results and the researcher judgement of which tools to use.	reviews might contribute to a more tailored treatment approach and eventually reduce pain intensity.<br><br>Further research is needed to consider more accurate measurement of neuropathic pain, which is difficult to assess in this population. Quantitative Sensory Testing could facilitate this.	

					were used to calculate the prevalence of pain. Logistic multinomial regression analysis for pain and dementia type differences.				
**Author, Date and Country**	**Aims**	**Condition**	**Sample/ description of population**	**Setting**	**Method**	**Assessment tool used**	**Main findings**	**Recommendatio ns**	**Authors conclusion**
Villanueva *et al* (2003) USA	To establish the reliability and validity of a measure to assess pain in individuals with advanced dementia	Advanced dementia	**Sample size 65** Study one; 25 residents (16 female, average age 84, 60% suffering from potentially painful conditions with 80% of these on analgesics, average level of dementia	Four long term care facilities (three skilled nursing facilities and one locked dementia assisted living facility). This included residents from	There were two studies; Study one assessed the reliability of PADE by calculating inter-rater reliability (by using two simultaneous raters and calculating the intraclass correlation coefficient ICC), internal	Pain Assessment for the Dementing Elderly (PADE), which was developed by the authors to help caregivers assess patient behaviour which may	Interrater reliability are well within acceptable ranges. Behaviour and facial expression can be reliably rated by observation. Internal consistency as assessed by Cronbach's alpha is acceptable and	The use of care staff (rather than research assistants) leads to recommendatio ns that this tool is clinically applicable. More research is needed to better understand the closely aligned factors of pain, agitation and	There is wide agreement that pain should be a standard part of medical and nursing assessment, particularly in this population. The PADE is a reliable and valid tool to assess pain in dementing elderly

			was 5.6 on the GDS). Study two; 40 residents (32 female, mean age 81.33, average level of dementia 5.28, 25% suffering potentially painful conditions with 27.5% of these on analgesics)	residential care facilities in four cities in study 1, and residents from one SNF in study 2. The setting is in Northwest USA	consistency (using Cronbach's alpha) and temporal stability (test re-test using ICC). Study two was to replicate the reliability findings of study one and assess the validity of PADE, by evaluating the relationship between PADE and a measure of agitation CMAI. The Mann-Whitney U test was used to assess criterion validity due to nonormal data. This study lacks detail	indicate pain. Study two also used the Cohen-Mansfield Agitation Inventory (CMAI)	suggests that PADE assesses a unified construct. Validity was also suggested by several factors. This adds to existing literature, suggesting a close relationship between agitation and pain. Pain is sometimes treated as agitation or other psychiatric difficulties and can be similarly interpreted by care home staff.	psychiatric symptoms.	residents of long-term care facilities, and can add to the clinical guidelines on pain assessment.

					regarding procedures used when carrying out the assessment in practice.				
Author, Date and Country	Aims	Condition	Sample/ description of population	Setting	Method	Assessment tool used	Main findings	Recommendations	Authors conclusion
Zwakhalen *et al* (2008) The Netherlands	The study aims to explore pain prevalence in nursing home residents with dementia using PACLAC-D to identify the association between pain, dementia and demographic parametres	Dementia (the majority being severe)	**117 residents** met the inclusion criteria. 80% female, mean age 82.8, mean MMSE score 5.7 (advanced dementia), most had Alzheimer's Disease.	Three Dutch nursing homes, dementia special care units	Observational study design. Participants were observed by 10 trained raters (caregivers and nurses) and assessed for pain during personal morning care. A step by step approach was conducted to determine the cut-off scores to indicate pain, tested using a logistic	PACSLAC-D; the Dutch version of the pain assessment checklist for seniors with limited ability to communicate. It is a shorter, revised version of PACSLAC of 24 items, established using principle component	The study showed that almost half the participants experienced pain but overall pain intensity scores were mild; the mean pain score of those who experienced pain was 6.31 out of 24. Those who experienced pain tended to be more cognitively	Observational pain scales such as the PACSLAC-D should help care workers recognise and evaluate pain cues in a more reliable, valid and simple manner. Further research is needed; a larger study on pain prevalence and using another staging instrument in addition to MMSE such as	This study confirms the expectation that pain prevalence in older residents with more advanced dementia in nursing homes is high.

					regression model. To examine inter-rater reliability, 15 residents were assessed by two raters simultaneously, analysed using intra-class correlation. Demographic information about participants was gathered, including type of dementia. Raters were unfamiliar with this information and with the resident, to make the assessment objective. A multiple linear regression	analysis and demonstrating high validity and internal consistency in previous studies.	impaired, and 40% of them did not receive any analgesia. Co-morbidities and analgesic use had the strongest associations were significantly related to pain, but there was not a significant difference between types of dementia. Depressive symptoms also had a weak association with pain, although depression was not optimally assessed so not a main	the GDS (the MMSE is mainly for screening). A higher number of raters would further explore inter-rater reliability of this tool. This area of research would also benefit from further investigation of the potential association between pain and depression.	

					model was used with pain as the outcome variable, and independent variables included type of dementia, medication and depressive symptoms.		outcome measure.		
**Author, Date and Country**	**Aims**	**Condition**	**Sample/ description of population**	**Setting**	**Method**	**Assessment tool used**	**Main findings**	**Recommendations**	**Authors conclusion**
Zwakhalen *et al* (2012) The Netherlands	To investigate the feasibility of regular pain assessment using an observational scale in nursing home residents with dementia and determine interventions applied after diagnosing possible pain	Dementia with severe cognitive deficits	**Sample size n=22,** who met the inclusion and exclusion criteria from a random selection of 40 residents. Average age 80, 115 female and 7 male, participants often had	1 nursing home on the geriatric ward in the Netherlands	Exploratory descriptive observational study over a six week collection period. Pain was measured twice a week using PACSLAC-D in daily nursing home practice. Intervention undertaken as	PACSLAC-D and semi-structured interviews	The completion rate was high; of the 264 scheduled assessments, 90% were completed although datasheets were often incomplete or inaccurate. The datasheets were	PACSLAC-D is a clinically useful tool, as indicated by the high compliance rate and staff evaluations. However this did not result in the frequent use of pain relieving interventions. Further work is required to develop pain	Providing staff with adequate pain assessment tools alone is not sufficient to change pain management practices. Pain relieving interventions were not frequently applied, and were mainly

			multiple diagnoses (mean of four comorbidities), 27% had known pain complaints, 41% used pain medication and 68% used psuchotrophi c drugs		a result of the pain score (the cut off score was 4) were recorded on a datasheet. After week 3 and week 6 structured interviews were conducted with staff members to evaluate the implementatio n of pain assessment. The study was conducted using the Medical Research Council framework on designing and evaluating multifactorial health interventions.		completed in 65% of cases and in approximately half these situations (with a score >4) no intervention was applied. Staff also reported interpreting pain cues was complicated and could be affected by reasons other than pain such as distress or panic. Data from interviews was summerised, rather than providing direct data/quotes, so reducing credibility. A	management plans and guidance to assist staff with pain knowledge, the conception of pain its treatment	non-pharmacologic al.

					Descriptive statistics were used for the demographic analysis and completion rates.		majority of interventions applied were non-pharmacological, such as comforting and distraction, as outlined on a table. This could be because of the low pain scores, but could also indicate a limited knowledge and misconception by staff about pain and its management.		
