# Supplementary material for: Pain Assessment for Individuals with Advanced Dementia in Care Homes: A Systematic Review
Source: Geriatrics (Basel). 2021 Oct 19;6(4):101. doi: 10.3390/geriatrics6040101 (PMC8544573; doi:10.3390/geriatrics6040101)
Supplement: Supplementary file 1 [file geriatrics-06-00101-s001.zip › geriatrics-1404136 supp/Table S3; Psychometric evaluation.pdf]

Table S3: Psychometric Evaluation of Pain Assessment Tools

Scale	Author	Items	Scoring	Interpretation	Item origin	Sample	Content	Criteria	Construct 1	Construct 2	Homogeneity	Between Observers	Test Retest	Applicability	Total
APS	Neville et al 2014 [34]	6	0-18	0-2=no pain, 3-7=light pain,8-13=moderate 14+=severe	2	2	2	0	2	2	2(alpha >0.7)	0	1	2	15
ADD	Lints-Martindale et al.2012 [30]	5	Not reported	Not reported	2	0	1	0	2	2	1(alpha 0.7)	2	0	Not reported	9
CNPI	Lints-Martindale et al.2012[30]	6	0-6	Not reported	1	0	1	0	2	2	0 (alpha 0.54)	2	0	Not reported	8
	Neville et al. 2014 [34]	6 items (rest v.movement)	0-6	Presence of any of the behaviours may indicate pain	1	2	2	0	2	1	2(alpha >0.7)	0	0	1	11

DisDAT	Jordan et al.2011 [29]	A creation of a list of signs and behaviours when distressed or content/neutral	The tool has not been designed to be scored	n/a	1	1	1	0	2	1	0	0	0	1	7
Doloplus-2	Neville et al. 2014 [34]	10 items, divided into 3 sub-groups	0-30	Each item is scored 0-3. Pain is patent for an overall score of 5 or greater	1	2	2	0	2	2	2(alpha >0.7)	0	1	2	14
e-PAT	Atee et al. 2018 [17]	42 items across 6 domains	0-20	Categorical ratings are 0-6 no pain, 7-11 mild pain,	2	0	2	0	0	0	2(alpha 0.925)	1	2	1	10

				12-15 moderate pain, 16+ severe pain											
	Hoti et al. 2018 [25]	Pain assessed at rest and movement across 6 domains	0-20	0-6 no pain, 7> pain	2	0	1	0	2	2	0	0	0	2	9
FACS	Hadjistavropoulos et al. 2018 [24]	Facial movements are divided into 44 possible action units.	Facial action units scored 0-5, and intensity scores 0-16	0 = no facial action to 5=maximum facial action.	1	1	1	0	0	2	0	2	0	2	9
MPS	Mahoney et al.2008 [31]	8	0-3	0=minimum pain to 3=severe pain	2	2	2	0	2	2	2 (alpha= 0.76)	1	1	1	15

MOBID	Husebo et al. 2009 [26]	5 active move ments, observ ing 3 pain behavi our indicat ion and scores the pain intensi ty	0-10	0-10 NRS scale used (o=no pain to 10=the worst pain possible)	2	0	1	0	0	2	0	2	2	1	10
MOBID-2	Husebo et al. 2014 [27]	5 active move ments(MOBI D-2 part 1) and intern al organs , head and skin (MOBI	Pain intensity 0-10	0=no pain, 10=as bad as it possibly could be	1	2	1	0	0	2	0	0	2 (active movem ents) 1(head, mouth, neck)	2	10

		D-2, part 2)													
NOPAIN	Lints-Martindale et al. 2012 [30]	4 parts	0-35	Pain was rated in intensity using a six-point Likert scale	1	1	1	0	2	2	2	2	1	1	13
PADE	Lints-Martindale et al. 2012 [30]	24 in 3 sections, but only 1 section used due to nature of the study	0-56	Not reported	1	1	1	0	2	2	2(alpha 0.76-0.88)	1	1	Not reported	11
	Villanueva et al. 2003 [40]	24 in 3 sections (Physical, Global Assessment and Functional)	0-56	Rating 1-4 for each item, denoting higher scores as higher distress (section 1), higher	1	1	1	0	0 (it is compared to an agitation scale)	1	1	2	1	1	9

				pain intensity (section 2) and less independence (section 3)											
PACSLAC	Cheung et al. 2008 [18]	60 items across 4 dimensions	0-60	Not reported	2	2	2	0	0	0	0	2	0	1	9
	Lints-Martindale et al. 2012 [30]	60 items across 4 dimensions	0-60	The presence of pain related behaviours is marked next to each item	2	1	2	0	2	2	2(alpha 0.83)	2	0	2	15
PACSLAC II	Hadjistavropoulos et al.2018 [24]	31 pain behaviours that correspond	0 or 1	0=absent 1=present	2	1	2	0	1	2	1(alpha =0.8)	2	0	2	13

		to the AGS scale													
PAIC	Van Dalen-Kok et al.2019 [38]	3 domains with 36 items			1	0	2	0	0	1	0	1 (70% agreement)	1	0	6
PAINAD	Jordan et al.2011 [28]	5	0-10	0-10 with higher scores indicating higher pain intensity	2	1	2	0	0	1	0	0	0	1	7
	Lints-Martindale et al. 2012 [30]	5	0-10	Not reported	2	1	1	0	2	2	2(alpha 0.73)	2	0	2	14
PAINE	Cohen-Mansfield et al. 2006 [19]	22 items	The first 15 items rated 0-7, and the remaining 7 items on a binary	7 point frequency scale from never to several times an hour	2	1	2	0	Informant rating 1-2 (moderate to high correlations) Observational data 0 (low correlation)	2	2	2	1	1	14

			yes/no scale												
